# Innate but Not Adaptive Immunity Regulates Lung Recovery from Chronic Exposure to Graphene Oxide Nanosheets

**DOI:** 10.1002/advs.202104559

**Published:** 2022-02-15

**Authors:** Thomas Loret, Luis Augusto Visani de Luna, Alexander Fordham, Atta Arshad, Katharine Barr, Neus Lozano, Kostas Kostarelos, Cyrill Bussy

**Affiliations:** ^1^ Nanomedicine Lab Faculty of Biology, Medicine and Health The University of Manchester Manchester Academic Health Science Centre Manchester M13 9PT UK; ^2^ National Graphene Institute The University of Manchester Manchester M13 9PL UK; ^3^ Lydia Becker Institute of Immunology and Inflammation Faculty of Biology, Medicine and Health The University of Manchester Manchester Academic Health Science Centre Manchester M13 9PT UK; ^4^ Catalan Institute of Nanoscience and Nanotechnology (ICN2) CSIC and The Barcelona Institute of Science and Technology (BIST) Campus UAB Bellaterra Barcelona 08193 Spain

**Keywords:** adaptive immunity, chronic exposure, graphene oxide, innate immunity, lungs, nanomaterials

## Abstract

Graphene has drawn a lot of interest in the material community due to unique physicochemical properties. Owing to a high surface area to volume ratio and free oxygen groups, the oxidized derivative, graphene oxide (GO) has promising potential as a drug delivery system. Here, the lung tolerability of two distinct GO varying in lateral dimensions is investigated, to reveal the most suitable candidate platform for pulmonary drug delivery. Following repeated chronic pulmonary exposure of mice to GO sheet suspensions, the innate and adaptive immune responses are studied. An acute and transient influx of neutrophils and eosinophils in the alveolar space, together with the replacement of alveolar macrophages by interstitial ones and a significant activation toward anti‐inflammatory subsets, are found for both GO materials. Micrometric GO give rise to persistent multinucleated macrophages and granulomas. However, neither adaptive immune response nor lung tissue remodeling are induced after exposure to micrometric GO. Concurrently, milder effects and faster tissue recovery, both associated to a faster clearance from the respiratory tract, are found for nanometric GO, suggesting a greater lung tolerability. Taken together, these results highlight the importance of dimensions in the design of biocompatible 2D materials for pulmonary drug delivery system.

## Graphical abstract

This work highlights the need to interrogate both innate and adaptive immunity to understand the impact of materials. Only the innate immunity is involved in the response to chronic pulmonary exposure to graphene oxide (GO) nanosheets, whereas multi‐walled carbon nanotubes activate both innate and adaptive immunity, and lead to fibrosis. Amongst all tested materials, nanometric GO has the safest profile.

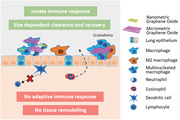

## 1 Introduction

Graphene has been extensively investigated for multiple purposes, ranging from electronic devices to water filtration, through composite materials or coating. In the biomedical field, graphene has also been proposed for an array of applications. This includes the production of scaffolds for tissue regeneration^[^ ^1^ ^]^ or the design of next‐generation implants,^[^ ^2^ ^]^ as well as the development of photo‐thermal therapy, vaccine platforms, or drug delivery systems.^[^ ^3^, ^4^, ^5^ ^]^ In this latter application, graphene oxide (GO), the oxidized form of graphene, has stood out due to two important features, namely a high surface area for high drug payload and a surface rich in oxygen groups. Having surface functional head‐groups increases the possibility of further chemical functionalization, which allows the design of nanocarriers with greater specificity.^[^ ^6^ ^]^ It also provides superior colloidal stability compared to other graphene nanomaterials. Regardless of the formulation, good colloidal stability is essential for the administration of drug delivery systems.

Whilst the potential benefits of GO to the biomedical field continue to be revealed, questions are concurrently raised regarding its safety profile and long‐term fate in the body. In several studies, limitations to GO biocompatibility have been identified and associated to the physicochemical features of the tested materials. In particular, variation in the size of GO sheets, ranging from few nanometers to micrometers, in thickness from single layer sheets to multi‐layer platelets, in degree of surface oxidation, and in durability under biological conditions were ascribed to potential safety limitations. To address these concerns, comprehensive investigations of the biocompatibility of GO within the intended application,^[^ ^7^, ^8^ ^]^ alongside the demonstration of its therapeutic efficacy, appears critical when developing new GO based drug delivery systems. This is particularly important when the intended route of administration is the respiratory tract. Inhalation is not only a promising route of administration for nanomedicine drug formulation in difficult‐to‐cure infections,^[^ ^9^, ^10^, ^11^ ^]^ but also a major route of unintended pulmonary exposure to engineered nanomaterials, which have raised serious safety concerns in the last 15 years.^[^ ^12^ ^]^

In this respect, one of the main concerns around GO and other graphene based materials comes from their proximity to the chemical composition of other carbon nanostructures, namely carbon nanotubes (CNTs) that have been reported to cause major lung issues. After inhalation, some CNTs were found to cause persistent inflammation, impairment of immune response,^[^ ^13^, ^14^ ^]^ formation of irreversible fibrotic lesions,^[^ ^15^, ^16^, ^17^ ^]^ necrosis,^[^ ^18^ ^]^ or even pulmonary carcinogenesis.^[^ ^19^ ^]^ In many of these reports, the extent of chronic immune response as well as the activation of the adaptive immunity caused by the tested CNTs were shown to be a hallmark in the progression toward pathological conditions.^[^ ^14^ ^]^ In addition, it was highlighted that the exact physicochemical characteristics of the tested materials (length, diameter, aspect ratio, rigidity, crystallinity, or nature of metal catalyst) and their ability to agglomerate^[^ ^20^ ^]^ and bio‐persist in the lungs were strongly associated to the extent of their impact.^[^ ^21^, ^22^, ^23^ ^]^ Solutions to mitigate these safety limitations have emerged from investigations in the chemical engineering and medicinal chemistry fields. Nevertheless, recurrent reports of major pulmonary adverse effects for some CNTs have weakened the promotion of other CNTs for biomedical application.

With this in mind, GO sheets may present several benefits over CNTs and could be a safer option for the pulmonary delivery of therapeutic molecules. Indeed, GO sheets do not possess a fibre‐shaped structure and can be produced as highly stable colloidal suspensions that are free of endotoxins and metallic impurities.^[^ ^24^, ^25^ ^]^ GO sheets have also been reported to either be eliminated rapidly or biodegraded under various physiological conditions.^[^ ^26^ ^]^ Functionalized forms of GO were even used successfully for intranasal immunization against influenza viruses in mice.^[^ ^11^ ^]^ However, these material‐related advantages do not preclude the ability of GO to cause adverse effects in the respiratory tract. Duch et al. reported that GO induced severe and persistent inflammation in mouse lungs over 21 days. They suggested that improved dispersibility to prevent agglomeration and low degree of oxidation were key to minimize the adverse effects.^[^ ^27^ ^]^ Consistent with these findings, Li et al. found that GO with higher degree of oxidation induces higher levels of lung inflammation.^[^ ^28^ ^]^ In another study, the agglomeration of GO sheets in the lungs was revealed as the main cause of acute lung injury leading to chronic inflammation and pulmonary fibrosis.^[^ ^8^ ^]^ In contrast, Bengston et al. reported that GO could agglomerate in mouse lungs following oropharyngeal aspiration and induce acute inflammation with hyperplasia, but not fibrosis.^[^ ^29^ ^]^ However, exposure to GO aerosols, which allow better representation of what a real‐world inhalation would cause, did not induce significant inflammation or any other major negative impact in rat lungs for up to 21 days.^[^ ^30^ ^]^ Our group has also previously investigated the pulmonary impact of GO.^[^ ^31^ ^]^ Following single intranasal instillation in mice, we showed that micrometric GO sheets induced pulmonary inflammation with granulomas that did not lead to fibrosis, but persisted for up to 90 days, whereas lungs exposed to smaller GO sheets recovered fully within 28 days. These clear size dependent differences between two GO types highlighted that materials designed with adequate lateral dimensions favoring better dispersibility could present safer profile in general and for intended application such as drug delivery. However, this study did not address the potential impact of a chronic exposure or the possible activation of the adaptive immunity after repeated exposure, although these two features were deemed significant in the evolution toward pathological conditions for CNTs. Generating such data would be extremely valuable toward the development of GO materials for pulmonary drug delivery.

The aim of the present study was therefore to assess the extent of lung tolerability of GO when successive pulmonary exposures were applied. Knowing that a single exposure to nanometric GO sheets could be well managed by physiological processes,^[^ ^32^ ^]^ we hypothesized that nanometric GO sheets would still demonstrate good tolerability due to a better clearance pattern and despite the repeated exposure regimen. On the contrary, micrometric GO sheets would trigger cellular and molecular pathways due to their longer lung residency confirming their inadequacy as candidate platform for pulmonary delivery. To test this hypothesis, a thorough immunological evaluation of the impact of these two types of GO was performed, using two doses of endotoxin‐free materials, and for up to 3 month after the last exposure. This was completed by a detailed histological analysis and evaluation of the materials’ clearance patterns using confocal Raman microscopy. The findings confirmed the hypothesis: micrometric GO sheets are not suitable platforms for pulmonary drug delivery as they induced widespread and long‐lasting tissue responses, despite noticeable recovery of the lungs and absence of adaptive immune response. In contrast, we found that nanometric GO sheets were relatively well tolerated by lung tissues, which recovered more rapidly owing to a faster clearance pattern involving innate immune cells only. Overall, these findings suggested that nanometric GO were eligible materials for exploring their potential as pulmonary drug delivery systems. Nevertheless, we would recommend administering a sub‐threshold low dose of nanometric GO to ensure the absence of adverse effects, guaranteeing the safety of the drug delivery system.

## 2 Results and Discussion

**Figure 1:**
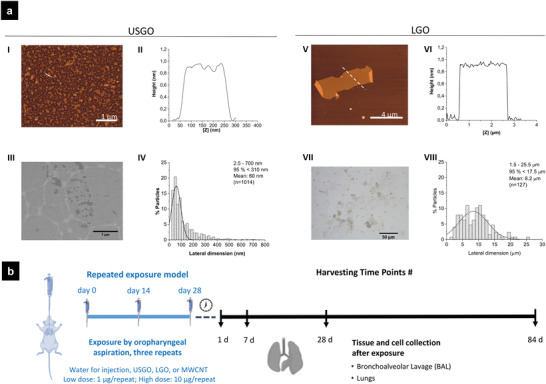
a) Structural and morphological characterization of USGO and LGO. I,V) Height atomic force microscopy (AFM) images; II,VI) height mapping along the dotted lines in (I) and (V) representative height atomic force microscopy (AFM) images, with (II) and (VI) height mapping along the dotted lines present in (I) and (V) cross sections height AFM images; (III) representative scanning electron micrograph of USGO sheets with (IV) corresponding lateral dimension distribution; (VII) representative optical microscope image of LGO sheets with (VIII) corresponding lateral dimension distribution. b) Repeated exposure experimental design. Mice (*n =* 6) were exposed by repeated oropharyngeal aspiration to ultra‐small graphene oxide (USGO), large graphene (LGO) oxide, multi‐walled carbon nanotubes (MWCNTs), or control vehicle (sterile water for injection). Animals were exposed once every 2 weeks to 1 or 10 µg of nanomaterials, with a total of three exposures, to achieve a final dose of 3 µg (low dose scenario) or 30 µg (high dose scenario). On days 1, 7, 28, or 84 after the last exposure, mice were euthanized, bronchoalveolar lavages (right lung only) were performed, and lungs were collected for further analysis. Left lungs were collected for flow cytometry (*n =* 3) and histology study (*n =* 3).

As mentioned above, GO physicochemical characteristics could be advantageous in the prospect of developing drug delivery systems for pulmonary administration.^[^ ^10^, ^11^ ^]^ Nevertheless, some questions remain unanswered regarding GO's safety profile. In particular, the long‐term impact and fate of these materials in the lungs is not well understood. To address these questions, mice were exposed by oropharyngeal aspiration to 1 or 10 µg of GO materials once every 2 weeks for 4 weeks, for a total administered dose of 3 or 30 µg per animal. GO sheet suspensions that contained either nanometer‐sized thin flakes (USGO) or larger micrometer‐sized (LGO) thin flakes (**Figure** **1**a) were used. Except the lateral size, the characteristics of the GO sheets were identical (i.e., thickness, surface characteristics (oxidation level, surface charge), lack of endotoxins, etc.) (Figure 1a; Table S1, Supporting Information).^[^ ^32^ ^]^ Moreover, the GO materials were produced following strict endotoxin‐free conditions. For enhanced comparison, reference, multi‐walled CNTs (MWCNTs; Mitsui‐7) were used as positive control due to their well‐known pulmonary adverse effects.^[^ ^33^, ^34^ ^]^ The MWCNTs were depyrogenated prior to administration in mice. A recovery time of 2 weeks between each exposure was chosen to evaluate a potential activation of the adaptive immunity (Figure 1b). The acute and chronic pulmonary adverse effects as well as the mechanisms involved in the biological response were investigated at days 1, 7 (1 week), 28 (1 month = 4 weeks), and 84 (3 months = 12 weeks) after the last exposure.

### 2.1 GO Caused a Transient Innate Immune Response and Preferentially Activated Macrophages toward Anti‐Inflammatory Subsets

#### 2.1.1 Influx of Granulocytes in the Airways

**Figure 2:**
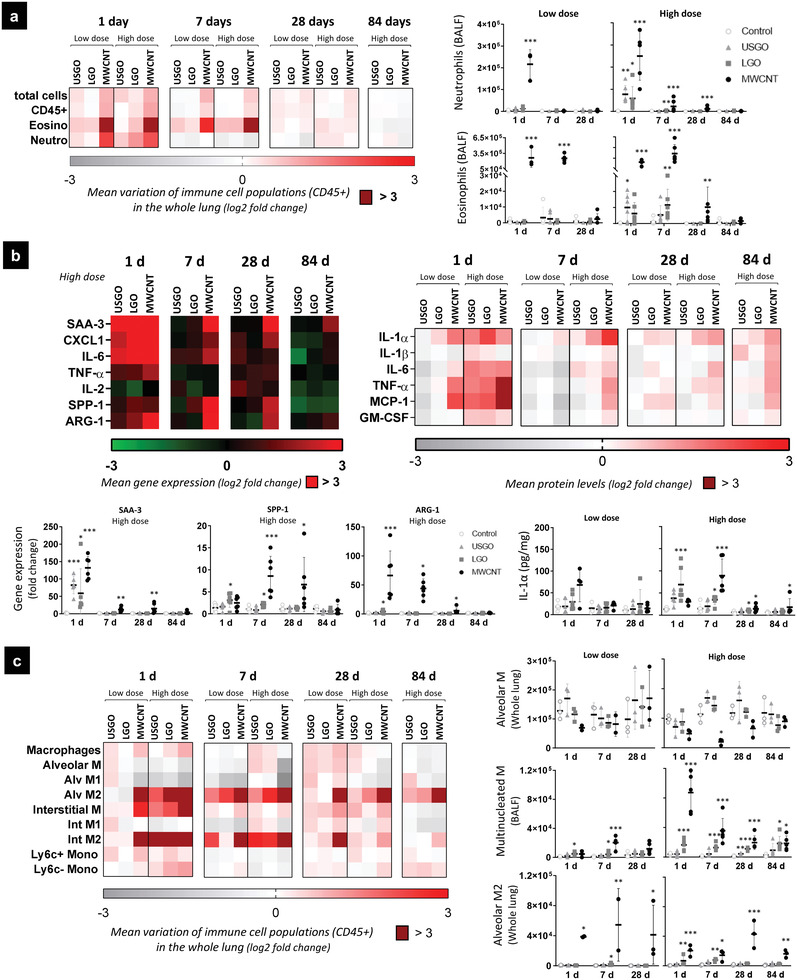
Innate immunity activation in response to repeated exposure to GO and MWCNTs. Whole lung samples and broncho‐alveolar lavage fluids (BALF) were analyzed to evaluate innate immunity activation. a) Phenotyping of granulocytes in the whole lung (flow cytometry) and BALF (colorimetric staining). b) Gene expression (RT‐qPCR) and protein levels (ELISA) in the whole lung. c) Phenotyping of macrophages and monocytes in the whole lung (flow cytometry) and BALF (colorimetric staining). For cell phenotyping in BALF, gene expression, and protein concentration, one‐way ANOVA followed by Dunnett's post‐hoc test or Kruskall–Wallis followed by Dunn's post‐hoc test was used to evaluate significant differences compared to the negative control for each time‐point (*n =* 6; ^*^ *p* < 0.05, ^**^ *p* < 0.01, and ^***^ *p* < 0.001). Two‐way ANOVA followed by Dunnett's post‐hoc test was used to evaluate changing in the number of cells in the whole lung (*n =* 3; ^*^ *p* < 0.05, ^**^ *p* < 0.01, and ^***^ *p* < 0.001).

Repeated exposure to endotoxin‐free GO sheets caused dose‐dependent acute inflammation, with influx of neutrophils and eosinophils in the alveolar space, irrespective of the sheet lateral dimensions (**Figure** **2**a; Figures S1,S2, Supporting Information). Differences were observed between the two GO materials; but overall the response was significantly lower in GO exposed animals compared to the MWCNT group. In the alveolar space, eosinophils were found to persist for a longer period of time than neutrophils, as they were observed for up to 7 days in GO materials groups and for up to 28 days in MWCNT exposed animals. A peak in eosinophils was noted at day 7 for LGO and MWCNTs but not for USGO. Noticeably, we observed a clear dose‐response for both GO types, but not for MWCNTs, although the recovery was faster at the lowest dose for MWCNTs. Differences were observed between bronchoalveolar lavages (BAL) and whole lung analyses (Figure 2a; Figures S1,S2, Supporting Information), showing that evaluating the specific influx of granulocytes in the alveolar space (i.e., BAL) was more sensitive.

#### 2.1.2 Activation of Pro‐Inflammatory Mediators in the Lungs

Concomitantly to the influx of granulocytes, a dose‐dependent increase in pro‐inflammatory mediators was noted at day 1 for all the materials (Figure 2b; Figure S3, Supporting Information). Interestingly, we found similar pattern of responses between USGO and LGO, with a peak at day 1 in both cases. At the highest dose, global inflammation at day 1 was slightly stronger for USGO compared to LGO, as highlighted by a higher expression in acute phase protein Serum Amyloid A3 (SAA3) (Figure 2b; Figure S3, Supporting Information). However, SAA3 levels were back to normal after 7 days, highlighting the absence of strong chronic inflammation, and a limited systemic impact following exposure to both USGO and LGO. At the highest dose, there was also a significant increase in gene expression or protein levels of the pro‐inflammatory cytokines IL‐1*α*, IL‐6, and TNF‐*α* for the two GO materials. Interestingly, an increased expression of CXCL1 was observed at day 1 (statistically significant for LGO but not USGO), underlining possible involvement of non‐immune cells in the inflammatory response to GO. Pro‐inflammatory mediators, including IL‐1*α*, were still significantly activated at days 7 and 28 for LGO but not USGO, suggesting a faster tissue recovery for USGO. In comparison, MWCNTs elicited a stronger and more long‐lasting response, involving significant chronic activation of pro‐inflammatory mediators. Several pro‐inflammatory markers were indeed still significantly upregulated and produced after 28 days (i.e., SAA3, CXCL1, and TNF‐*α*) or 84 days (i.e., IL‐1*α* and IL‐6) (Figure 2b; Figure S3, Supporting Information). Noticeably, a clear dose‐response was observed for MWCNTs, with significant increases in pro‐inflammatory markers at days 7 and 28 at the high dose but not the low dose. This difference between doses could explain why a significant increase of neutrophils in the alveolar space was observed until day 28 at the high dose only (Figure 2; Figure S2, Supporting Information).

#### 2.1.3 Impact on Lung Macrophage Populations

In whole‐lung samples, a dose‐dependent replacement of alveolar macrophages by interstitial ones was observed for all materials (Figure 2c; Figure S4, Supporting Information). This aligns well with the significant increase in the monocyte chemoattractant mediator MCP‐1 found in the lungs of both GO and MWCNT exposed animals (Figure 2b; Figure S3, Supporting Information). This replacement has been previously observed under acute and chronic inflammatory conditions.^[^ ^35^ ^]^ However, we did not observe activation of macrophages toward pro‐inflammatory subsets (M1), even at day 1 after the last exposure. Instead, we noted an activation toward alternative phenotypes (M2) that was dose‐dependent for GO but not for MWCNTs. At the high dose, we observed increases in interstitial M2 macrophages that were significant at day 1 for both USGO and LGO (Figure S4, Supporting Information). Significant increases in anti‐inflammatory alveolar M2 macrophages were observed up to 7 days for the LGO but not for the USGO compared to the control (Figure 2c). A clear dose‐dependent increase in multinucleated macrophages, suggesting fusion of macrophages, was also observed in BAL fluids for up to 84 days in LGO exposed animals (Figure 2c). Fusion of macrophages with other macrophages or immune cells is typically observed in presence of foreign bodies and aims to eliminate large particles more efficiently.^[^ ^36^ ^]^ Their presence at 28 days for USGO and up to 84 days for LGO, in absence of significant arginase expression (Figure 2b), suggests a shift of macrophage phenotype over time and highlights the importance of macrophage plasticity for the regulation of the immune response.^[^ ^37^, ^38^ ^]^ In comparison to GO materials or the vehicle control, repeated exposure to MWCNTs induced a greater activation toward M2 alternative phenotypes (Figure 2c). MWCNTs caused also a higher and dose‐independent increase in arginase positive macrophages compared to the vehicle control. A significant increase in these macrophages was still observed 84 days after the last exposure to MWCNTs at the highest dose.

Acute pro‐inflammatory responses, involving cells of the innate immunity, were shown in previous studies after single oro‐pharyngeal aspiration to GO^[^ ^29^ ^]^ or other graphene‐based materials.^[^ ^39^ ^]^ However, the present study provides new evidences that chronic exposure to endotoxin‐free GO could preferentially activate lung macrophages toward alternative subsets. Similar M2 activation has been previously reported after intraperitoneal injection of GO^[^ ^40^ ^]^ or graphene quantum dots,^[^ ^41^ ^]^ and after pulmonary exposure to CNTs.^[^ ^14^ ^]^

### 2.2 GO Did Not Significantly Activate the Adaptive Immunity

The activation of macrophages toward anti‐inflammatory subsets in GO exposed animals agreed well with the presence of granulomas^[^ ^42^ ^]^ at the high dose (Table S2, Figure S5, Supporting Information) or the activation of inflammatory mediators linked to granulomas,^[^ ^43^ ^]^ including osteopontin (SPP‐1) until day 7, and IL‐1*α* until day 28 in LGO exposed animals (Figure 2b). The presence of granulomas, along with the transient apparition of small bronchus‐associated lymphoid tissue (BALT) structures in some but not all animals exposed to GO (Table S2, Figure S5, Supporting Information), could suggest an involvement of lymphocytes in the immune response caused by repeated exposure to GO.

#### 2.2.1 Impact of GO on Lymphocyte Populations and Activation of Th1, Th2, and Th17 Pathways

**Figure 3:**
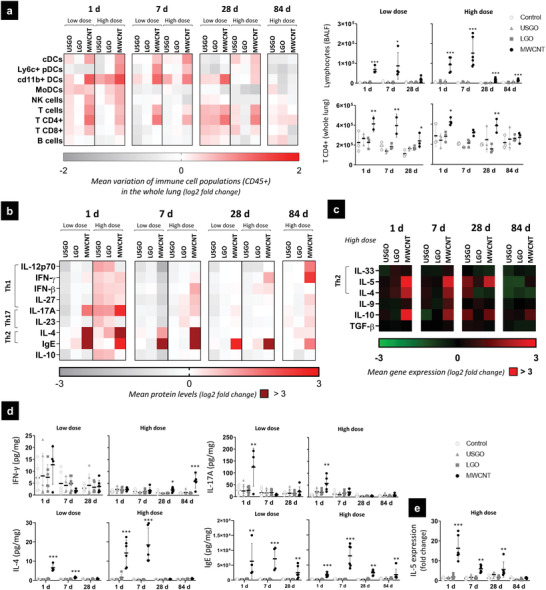
Adaptive immunity activation in response to repeated exposure to GO and MWCNTs. Whole lung samples and broncho‐alveolar lavage fluid (BALF) were analyzed to evaluate adaptive immunity activation. a) Phenotyping of dendritic cells and lymphocytes in the whole lung (flow cytometry) and BALF (colorimetric staining). b,d) Protein levels (ELISA) and c,e) gene expression (RT‐qPCR) of inflammatory mediators associated to adaptive immunity. For cell phenotyping in BALF, gene expression and protein levels, one‐way ANOVA followed by Dunnett's post‐hoc test or Kruskall–Wallis followed by Dunn's post‐hoc test was used to evaluate significant differences compared to the negative control for each time‐point (*n =* 4–6; ^*^ *p* < 0.05, ^**^ *p* < 0.01, and ^***^ *p* < 0.001). Two‐way ANOVA followed by Dunnett's post‐hoc test was used to evaluate changing in the number of cells in the whole lung (*n =* 2–3; ^*^ *p* < 0.05, ^**^ *p* < 0.01, and ^***^ *p* < 0.001).

There was no evidence of lymphocyte recruitment, as highlighted by the absence of significant increase in CD4^+^ or CD8^+^ cell populations in the whole lung, or the absence of increase in total lymphocytes in the alveolar space (**Figure** **3**a; Figure S6, Supporting Information). There was also no activation of type 1 inflammation, as no significant increases in IFN‐*γ* or the IL‐12 family cytokines were measured in the lungs after exposure to GO (Figure 3b; Figure S7, Supporting Information). In contrast, we found increases in protein levels of TNF‐*α* (significant only for LGO; Figure 2b;Figure S3b, Supporting Information) and IL‐17A (non‐significant; Figure 3d) for both GOs at day 1 and the highest dose. Although these two cytokines can be released by Th1 or Th17 lymphocytes, respectively, other cells can be involved in their secretion, including many cell types for TNF‐*α*, and neutrophils or macrophages for IL‐17A.^[^ ^44^, ^45^ ^]^ Moreover, we did not measure significant increases in production or expression of type 2 inflammation mediators (e.g., IL4, Figure 3b,c) or immunoglobulin E (IgE) (Figure 3b,d) after exposure to GO, although some eosinophils were found in the lungs (Figure 2a). Taken together, these findings support the idea that there was no activation of the adaptive immunity or any allergy‐like response in GO exposed animals. In addition, despite a transient increase in M2 arginase positive macrophages (Figure 2c), we did not observe any significant increase in IL‐10 or TGF‐*β* (Figure 3b,c; Figure S7, Supporting Information), suggesting that their presence may not be related to a sustained Th2 pathway.^[^ ^46^, ^47^ ^]^ Interestingly, we found an increase in non‐mature resident dendritic cells (CD11b^+^ DCs) at day 1, but only for LGO and at the highest dose, and with no increase in conventional DCs (cDCs) (Figure 3a; Figure S8, Supporting Information). This absence of increase in cDCs also supports our conclusions that pulmonary exposure to GO sheets does not significantly activate adaptive immunity.^[^ ^48^ ^]^

#### 2.2.2 Comparison of GO and MWCNTs Impact on Adaptive Immunity

In contrast to GO, lymphocytes were involved in the immune response to MWCNTs, used here as positive control for adverse effects (Figure 3a; Figure S6, Supporting Information). A significant increase in lymphocytes in BAL fluids and distinct BALT structures were found at every time‐point for MWCNTs, with a peak at 7 days. Variations in specific populations were also observed in the lungs, including significant increases in NK cells at day 1 and T helper populations (CD4^+^) at days 1, 7, and 28. We also found an increase in cDCs after repeated exposure to MWCNTs (Figure S8, Supporting Information), suggesting that DCs maturation could have caused further activation of lymphocytes toward CD4^+^ Th2 subsets.^[^ ^48^ ^]^ Moreover, we measured a significant increase in expression or protein levels of the Th2 mediators IL‐4 and IL‐5 (Figure 3b–e) associated to a strong and chronic eosinophilia in the alveolar space (Figure 2a; Figure S2, Supporting Information). These results together with the increase in IgE and the observation of Charcot Leyden like crystals crystals^[^ ^49^ ^]^ in the bronchia (Figure 3b,d; Figure S5a–c, Supporting Information) were indicative of a Th2 response,^[^ ^46^ ^]^ as previously reported after single pulmonary exposure to MWCNTs.^[^ ^14^ ^]^ Interestingly, although we identified a dose‐dependent secretion of innate or adaptive inflammatory mediators into the lungs, the influx of immune cells, and the allergy‐like response, especially the recruitment of eosinophils and lymphocytes in the BAL fluids, were not dose‐dependent (Figures 2,3). This leads to the conclusion that the innate immune response to MWCNTs was dose‐dependent, whereas the adaptive immune response is independent from the dose, highlighting the greater health risk of these materials compared to GO. Indeed the absence of significant lymphocyte activation and adaptive immune response to GO sheets, irrespective of their lateral dimensions, constitutes a major difference compared to MWCNTs. This important difference demonstrates the relative immune tolerability of GO following repeated pulmonary exposure and highlights the safer profile of the material.

### 2.3 GO Did Not Cause Lung Tissue Remodeling and Showed Good Recovery

**Figure 4:**
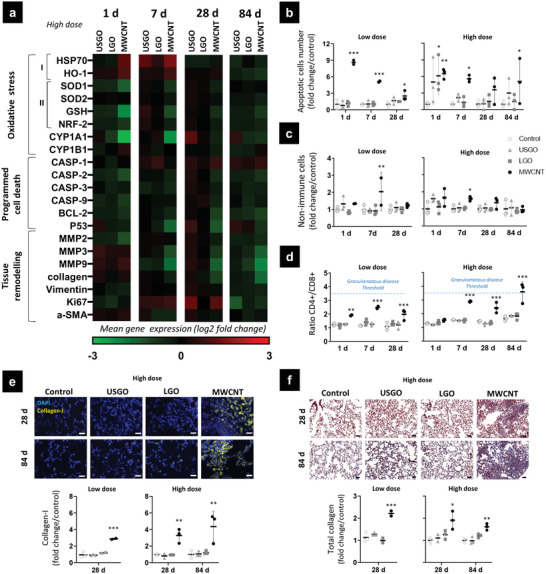
Mechanisms associated to oxidative stress, cell death, and tissue remodeling in lungs exposed to GO and MWCNTs. 1, 7, 28, and 84 days after the last exposure, lungs were harvested and processed for analysis of a) gene expression in right lungs by RT‐qPCR (*n =* 5–6), of oxidative stress I) pro‐oxidant, II) anti‐oxidant, programmed cell death, and tissue remodeling markers. b) TUNEL assay performed in left lung sections to investigate relative apoptosis over‐time (*n =* 3). c) Number of non‐immune cells counted by flow cytometry in digested left lungs (*n =* 3). d) Ratio of CD4+/CD8+ lymphocytes evaluated by flow cytometry in digested left lungs (*n =* 3), dotted blue lines represent the granulomatous disease threshold based on existing literature.^[^ ^54^ ^]^ e) Semi‐quantitative analysis of collagen‐I deposition in lung sections at 28 and 84 days (*n =* 3). f) Lung sections stained with Masson Trichrome to evaluate collagen deposition at days 28 and 84 (*n =* 3). One‐way ANOVA followed by Dunnett's post‐hoc test or Kruskall–Wallis followed by Dunn's post‐hoc test were used to evaluate statistical differences compared to the negative control (^*^ *p* < 0.05, ^**^ *p* < 0.01, and ^***^ *p* < 0.001). Scale bar corresponds to 50 µm.

In the present study, none of the endotoxin‐free GO tested, regardless of the dosage applied, caused any visible damages to the alveolar or bronchial epithelium (Table S3, Figure S5, Supporting Information). Moreover, no evidence of collagen deposition was found in USGO or LGO exposed animals compared to the vehicle control (**Figure** **4**). In contrast, MWCNTs induced collagen deposition at 28 days irrespective of the dose applied (Figure 4f). Evidence of a time‐dependent downregulation of collagen gene expression (statistically significant at 28 and 84 days; Figure 4a) was also found, suggesting a feedback regulation of the gene expression as the protein is secreted and the lung fibrosis settled (Figure 4a; Figure S10, Supporting Information). Despite the collagen deposition observed in the lungs of MWCNT exposed animals, no significant alteration of the gene expression for *α*‐SMA and Vimentin (i.e., biomarkers of EMT process often associated to fibrogenesis^[^ ^50^, ^51^ ^]^) was found at any time‐point (Figure 4a; Figure S10, Supporting Information). However, an alveolar and bronchial cell hyperplasia in tissue sections, and a decrease in matrix metalloproteinase gene expression in whole tissue lysates were identified in MWCNT exposed animals (Figure 4a; Figures S5a,b, S10, Table S3, Supporting Information). These last results likely contributed to the formation of fibrotic scars and further confirmed the negative impact of MWCNT on lung tissue plasticity.

#### 2.3.1 Adaptive Immunity Activation and Progression toward Pathological Conditions

The absence of significant lymphocyte recruitment or chronic activation of Th2 mediators following GO exposure and the opposite following MWCNT exposure could explain both the rapid inflammation resolution found after repeated GO exposures and the lung tissue remodeling observed after repeated MWCNT exposures. Lung tissue remodeling is characterized by the occurrence of structural changes in the lung parenchyma or in the airways, including changes of epithelial tissue structure and morphology, or evidence of fibrosis.^[^ ^52^ ^]^ Indeed, chronic Th2 response with IL‐4 secretion, as observed for MWCNTs, has been shown to be critical for collagen deposition and the formation of fibrotic granulomas.^[^ ^46^, ^47^, ^53^ ^]^ By promoting chronic eosinophilia, epithelial cell proliferation and differentiation through inhibition of matrix metalloproteases and fibroblast activation, the activation of lymphocyte CD4^+^ toward Th2 subsets is critical in maintaining the type 2 polarization.^[^ ^46^ ^]^ This underline the importance of evaluating adaptive immunity activation to predict long‐term lung damages and evolution toward pathological conditions. In the present study, the formation of fibrosis and progression toward granulomatous disease after MWCNT exposure was further confirmed by the appearance of a late‐onset Th1 response at 84 days (e.g., increase of INF*γ*; Figure 3b,d; Figure S7, Supporting Information) and the increased CD4^+^/CD8^+^ ratio irrespective of the dose applied (Figure 4d).^[^ ^54^ ^]^ A Th1 response would typically be expected to occur in the acute phase of the response to MWCNT exposure.^[^ ^14^ ^]^ Nevertheless, its absence at early time‐points can be explained by the repeated administration regimen applied. Indeed, within the context of repeated exposures used here, it is rationalized that when the last (third) exposure was applied, resolution of inflammation and healing processes that follow the previous (second) exposure were still ongoing. Therefore the overall biological response after the last exposure will be a combination of acute inflammatory (likely Th1 like response) and maturation/healing/remodeling phases (dominantly Th2 like response). It may even be possible that long‐lasting effects due to the first exposure have contributed to the outcomes measured after the last exposure. Altogether, these combined responses (to each exposure) may explain the unconventional increase in specific Th2 markers measured here in the early phase of the response to the last MWCNT exposure (e.g., day 1) and the late‐onset Th1 response.

#### 2.3.2 Role of Macrophages in the Maintenance and Resolution of the Inflammation

In an inflammatory environment, the role played by macrophages is important for both the maintenance and the resolution of the immune response. Macrophages activated to anti‐inflammatory subsets can suppress inflammation mediated by M1 macrophages through the secretion of IL‐10 and TGF‐*β* to prevent installation of type 2 inflammation.^[^ ^55^ ^]^ On the contrary, tissue repair macrophages activated in a Th2 environment were shown to be important in maintaining a type 2 inflammation.^[^ ^46^, ^47^ ^]^ In the present study, these two macrophage population subsets were not distinguished but combined and identified as M2, based on their arginase expression (Figure 2c). As a result, both GO sheets and MWCNTs were found to promote M2 macrophages, despite obvious differences in the immune response following exposure to these two categories of materials. For MWCNTs, both M2 macrophages and Th2 mediators were increased. This suggests that macrophages were pushed toward the tissue repair subset. In contrast, considering the absence of a distinct Th2 response after exposure to GO, the results suggest that GO sheets were promoting a macrophage differentiation toward anti‐inflammatory subsets rather than tissue repair phenotypes. In addition, in the absence of Th2 activation, anti‐inflammatory macrophages have been reported to regulate the immune response, preventing the development of chronic inflammation or collagen deposition in the airways.^[^ ^46^ ^]^ This immune regulation by anti‐inflammatory macrophages would support our findings of a successful recovery after exposure to either LGO or USGO, and would explain lung tissue remodeling after exposure to MWCNTs (Figure 4; Figure S5, Supporting Information).

### 2.4 Biological Response to Pulmonary Exposure is Material‐Specific

GO sheets and MWCNTs present numerous physicochemical differences that can explain the discrepancies between the biological responses to these materials. The two intertwined structural parameters that apply to both GOs and MWCNTs and have been associated before to adverse effects are the aspect‐ratio and rigidity/flexibility.^[^ ^56^ ^]^ The MWCNTs used here were tubular nano‐objects with a typical diameter of 49 ± 13.4 nm for a length ranging between 1 and 20 µm (median = 3.86 µm).^[^ ^32^, ^57^ ^]^ In contrast, the GO sheets were thin 2D nano‐objects (below 10 nm thickness, vast majority ≈1 nm) with lateral dimensions ranging from 10–300 nm (for USGO) to 1–30 µm (for LGO), which gave them a platelet‐like shape.^[^ ^58^ ^]^ The lateral dimensions of the LGO sheets may qualify them as high aspect ratio materials to a similar extent as the MWCNTs. However, LGO sheets appeared to remain flexible nano‐objects (see videos in ref. [59] in which the same materials were used) compared to MWCNTs that behave like rigid needles.^[^ ^60^ ^]^

#### 2.4.1 Impact of the Aspect Ratio on the Biological Response to GO and MWCNTs

Long and rigid MWCNTs similar to those used here, owing to their fibre‐like aspect, were shown to cause specific adverse effects. Oxidative stress, immune cell death by piercing cell and lysozyme membranes,^[^ ^61^ ^]^ as well as granulomatous inflammation leading to fibrotic tissue and persistence in the lungs after pulmonary exposure have been extensively reported.^[^ ^62^ ^]^ In the present study, both significant increases in gene expression of the pro‐oxidative heat shock proteins HSP70 and HO‐1 at both days 1 and 7, and significant decreases in gene expression of the anti‐oxidant markers GSH, SOD1, and NRF‐2 at the earliest time‐points were found after repeated exposure to MWCNTs (Figure 4; Figure S9, Supporting Information). This is in agreement with the previous studies mentioned above, highlighting the oxidative stress caused by these well‐studied MWCNTs. Interestingly, significant inhibition in CYP1A1 was also identified (Figure 4; Figure S9, Supporting Information), suggesting a possible decrease in MWCNT detoxification processes, as reported for other carbon substances including benzo[a]pyrene.^[^ ^63^ ^]^ The downregulation of CYP1A1 expression could also be attributable to the presence of oxidative stress.^[^ ^64^ ^]^ Finally, although there was a dose‐independent increase in the number of apoptotic cells at all the time‐points after MWCNT exposure highlighting the toxicity of these materials (Figure 4b), there was a downregulation of two regulators of apoptosis namely caspase‐9 and ‐2 (Figure S11, Supporting Information). While evolution of mRNA levels should be interpreted with cautious because of post‐translational regulation (i.e., pro‐caspase vs active caspase), this decrease may explain the increase in non‐immune cell number found in the lungs at day 7 after MWCNTs (Figure 4c). Such increase after MWCNT exposure would be expected since Th2 immune response has been reported to cause hyperplasia of the epithelium.^[^ ^46^ ^]^ In contrast, GO sheets irrespective of their dimensions did not cause sustained oxidative stress (Figure S9, Supporting Information) or sustained apoptosis beyond day 1 at the highest dose (Figure 4b; Figure S11, Supporting Information). These two findings support the lack of adaptive immune response or lung tissue remodeling after GO exposure, and underline the safer profile of GO sheets compared to MWCNTs.

#### 2.4.2 Role of Metallic Impurities in the Biological Response to GO and MWCNTs

The presence of metallic impurities in nanomaterials has been associated to greater adverse effects in lungs. Pulmonary exposures to soluble metals or metallic particles have been shown to activate the adaptive immunity and trigger allergy‐like Th2 responses, causing tissue damages.^[^ ^65^, ^66^ ^]^ Carbon nanomaterials containing metallic catalyst particles may also release those impurities in the form of ions after internalization by immune cells.^[^ ^67^, ^68^ ^]^ This release of metallic ions from carbon nanomaterials, especially iron, has been associated to greater toxicity.^[^ ^69^, ^70^, ^71^, ^72^ ^]^ Importantly, we did not detect any metallic impurities in our in‐house GO sheets,^[^ ^32^ ^]^ while the MWCNTs used here are known to have metallic residues (see Table 1 in ref. [73]). Therefore, the lack of metallic impurities in GO materials and their presence in MWCNTs might explain the absence of Th2 response and lung tissue remodeling after repeated exposure to GO, and the dose‐independent allergy‐like response to MWCNTs. Further investigations remain necessary to evaluate the impact of metallic impurities presence in the toxicity of these nanomaterials. Nevertheless, such assessment is difficult since the use of purification methods also alters the physicochemical characteristics of the materials.^[^ ^34^ ^]^

### 2.5 GO Fate in the Lungs is Size‐Dependent

#### 2.5.1 GO Clearance from the Lungs

**Figure 5:**
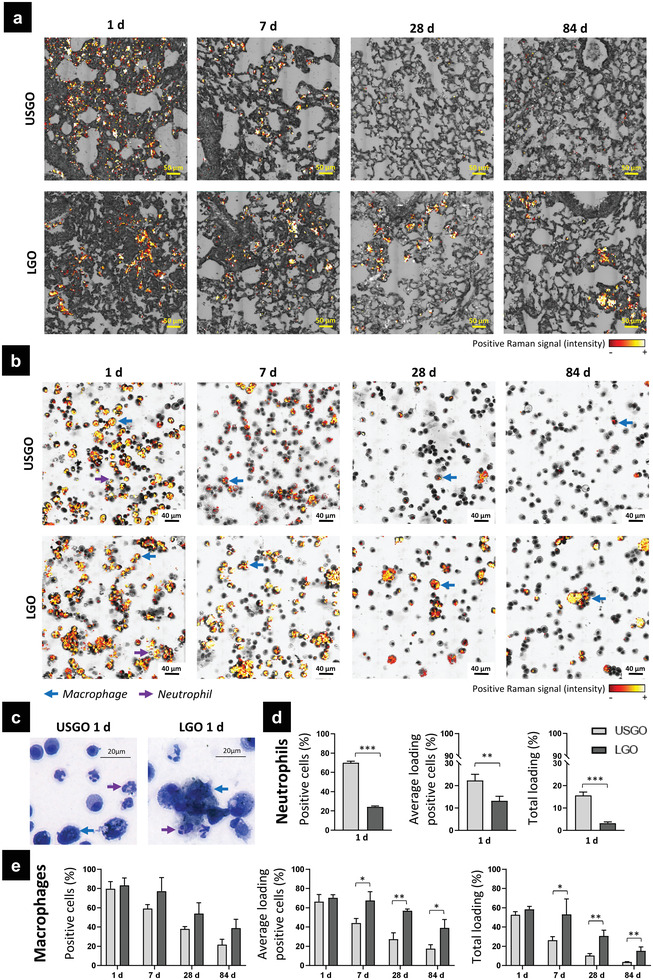
Size‐dependent clearance of GO materials. a) Lung sections and b) broncho‐alveolar lavage fluid (BALF) slides were analyzed by Raman spectroscopy imaging. BALF slides were then stained for cell phenotyping with Kwik–Diff staining usually used for blood cell morphology test to identify cells and evaluate material internalization by macrophages and neutrophils. c) High magnification images of BALF cells at day 1 after repeated exposure to USGO and LGO. d) Internalization of USGO and LGO in neutrophils at day 1. The proportion of neutrophils that have internalized GO (positive cells (%)), the average loading of GO‐positive cells (%) and the total loading (%) are presented. e) Internalization of USGO and LGO in macrophages at days 1, 7, 28, and 84. The proportion of macrophages that have internalized GO (positive cells (%)), the average loading of GO‐positive cells (%), and the total loading (%) are reported. At each time‐point, *t*‐test or Man–Whitney test were used to evaluate statistical differences (*n =* 3; ^*^ *p* < 0.05, ^**^ *p* < 0.01, and ^***^ *p* < 0.001).

When comparing LGO and USGO, most findings from the present study were pointing toward the activation of similar cellular and molecular mechanisms. Nevertheless, LGO sheets were inducing a longer pro‐inflammatory reaction and a stronger anti‐inflammatory response compared to USGO sheets. The reduced and shorten biological response to USGO exposure could be explained by a faster clearance from the lungs of USGO compared to LGO, as suggested by Raman spectroscopy‐based images (**Figure** **5**a). Indeed, while there were clear evidences of remaining materials in LGO exposed lungs after 84 days, it was difficult to identify remaining USGO materials at the same time point despite their presence at earlier time points. These findings agree with our previous study in which a time‐dependent and size‐dependent clearance of GO sheets from the lungs was also reported after single intranasal administration.^[^ ^31^ ^]^

#### 2.5.2 GO Internalization by Neutrophils

Going further in the understanding of the clearance process, we then identified that the recruited neutrophils, which were found in BAL fluid only at day 1 (Figure S1, Supporting Information), may play an essential role in the rapid elimination of USGO materials from the respiratory tract (Figure 5b). Indeed, Raman imaging coupled with differential staining of BAL cells from GO exposed lungs highlighted a greater interaction between neutrophils and USGO than with LGO; 70% of neutrophils in USGO condition were GO‐positive, while only 20% of neutrophils in LGO condition were GO‐positive. This difference in interaction was associated to a difference in the relative amount of materials per neutrophil (total loading), reaching 15.6% for USGO and only 3.2% for LGO. Material internalization by neutrophils has been previously reported in blood samples or in vitro.^[^ ^74^ ^]^ We therefore theorized that the greater interaction revealed by Raman could be evidence of a greater internalization of USGO sheets by the recruited neutrophils in comparison to LGO. In this hypothesis, the differences observed between USGO and LGO could be attributed to a size‐dependent internalization, with USGO nanosheets being more internalized due to smaller dimensions. Since neutrophils have the ability to reverse migrate quickly to lymph nodes or blood vessels as inflammation resolved,^[^ ^75^ ^]^ a greater amount of USGO than LGO could have been cleared from the lungs by reverse migration of GO laden neutrophils, reducing more quickly the overall lung burden for USGO in comparison to LGO.

#### 2.5.3 GO Internalization and Clearance by Alveolar Macrophages

When comparing different time points, Raman imaging of BAL cells also revealed a fast and continuous decrease in relative amount of USGO interacting with BAL macrophages (from 52.6% at 1 day to 3.7% at 84 days; Figure 5b). This was suggesting that migration of GO laden macrophages, likely relocating to draining lymph nodes or eliminated following mucociliary clearance mechanisms, may also play a significant role in USGO elimination from the respiratory tract alongside the reverse migration of GO laden neutrophils. In contrast, BAL macrophages in LGO exposed animals remained relatively unchanged for up to 28 days after the last exposure (58.4% at 1 day, still 30.6% at 28 days, then 15.1% at 84 days; Figure 5b). The persistence of LGO materials in lungs and BAL fluids could be explained by the relative inability of macrophages to internalize large materials, which in turn leads to macrophage fusion and decreased elimination. This aligns well with the findings of a greater amount of multinucleated macrophages after LGO exposure than after USGO exposure, especially at the high dose (Figure 2c). Moreover, the persistence of GO laden BAL macrophages in the case of LGO exposed animals was consistent with the greater number of M2 macrophages (Figure 2c) and granulomas (Table S2, Figure S5, Supporting Information), as well as the greater activation of specific inflammatory markers expressed in these granulomas (e.g., IL‐1*α*; osteopontin, SPP‐1) (Figure 2b). Indeed, macrophage fusion and activation toward M2 phenotypes, as well as the formation of granulomas, have been reported before when foreign bodies including discoid materials^[^ ^76^ ^]^ cannot be internalized efficiently by macrophages.^[^ ^36^ ^]^ Therefore, the large aspect ratio of the LGO sheets (i.e., platelet‐like shape and large lateral dimensions) may be a disadvantageous feature in respect to their clearance by resident and recruited macrophages,^[^ ^56^, ^77^ ^]^ or neutrophils, in comparison to USGO nanosheets.

In addition to a cell‐mediated clearance, the elimination of GO materials from the lungs (i.e., fast and constant for USGO, at slower pace for LGO) could also be attributed to material degradation. Both neutrophils and macrophages have indeed been reported to not only remove but also digest carbon nanomaterials.^[^ ^26^ ^]^ Further research looking specifically at the degradation of GO in laden neutrophils and macrophages, in the lungs, or after migration in secondary locations such as draining lymph nodes, are therefore warranted to reveal the final fate of GO sheets after pulmonary exposure. This is a key requirement for the clinical translation of promising pulmonary nanovectors.

## 3 Conclusions

In the present study, we comprehensively evaluated the lung tolerability of two types of endotoxin‐free GO materials with varying lateral dimensions in mice after repeated pulmonary exposure. The immune response and histology of the lungs were investigated for up to 3 months after the last exposure. We found that regardless of their lateral dimensions, GO sheets caused a dose dependent acute immune response involving primarily cells of the innate immunity. At later time points, there was no activation of the adaptive immunity or allergy‐like response for any of the GO materials. Instead, there were clear evidences of immune response resolution and lung tissue recovery for both GO materials, even at the highest dose. Lateral dimensions were found to influence the internalization of GO sheets by phagocytic cells and their persistence in the lungs, which in turn affect the pace of tissue recovery. While the nanometric GO materials were eliminated rapidly, consistent with a faster tissue recovery, the micrometric GO materials persisted in the lungs and caused longer lasting effects such as granulomas. Taken together, these results highlight the ability of the immune system to resolve the sterile inflammation caused by repeated pulmonary exposures to endotoxin‐free GO materials, irrespective of their lateral dimensions. They also demonstrate that reducing lateral dimensions improved the tolerability of GO in lungs owing to a better internalization by phagocytes leading to a better clearance profile. This comprehensive analysis of GO lung tolerability will help guiding the safe development of future pulmonary nanomedicines based on GO sheets, as well as providing wider stakeholders with key information regarding the pulmonary impact of GO‐based nanomaterials.

## 4 Experimental Section

### GO Materials

Biological‐grade USGO and LGO materials, dispersed in sterile water for injection, were produced by the modified Hummers’ method, under strict endotoxin‐free conditions as previously reported,^[^ ^30^ ^]^ using graphite powder as starting material (Merck‐Sigma). The two GO materials were then fully characterized (Table S1, Supporting Information) in a similar fashion to what was previously described.^[^ ^30^ ^]^ The structural and morphological properties of USGO and LGO sheets were determined by atomic force microscopy (AFM), scanning electron microscopy (SEM), and optical microscopy (Figure 1a). First, AFM images were acquired using an Asylum MFP‐3D atomic force microscope (Oxford Instruments) operating in standard air‐tapping mode and equipped with silicon probes (Ted Pella) with a resonance frequency of 300 kHZ; a nominal force of 40 N m^−1^ was used to characterize the surface. Samples were prepared by drop casting 20 µL of GO suspension (100 µg mL^−1^) onto a freshly cleaved mica surface (Ted Pella) previously covered with 20 µL of poly‐L‐lysine 0.01% solution (Merck‐Sigma), subsequently washed with water, and then dried overnight at room temperature. Images were processed using Gwyddion software (http://gwyddion.net, version 2.56). To obtain the lateral dimension distribution, analysis of height in AFM images was performed using ImageJ software (National Institutes of Health, USA, https://imagej.nih.gov). Second, SEM images were recorded on a Magellan 400L field emission scanning electron microscope (Oxford Instruments), which was equipped with an Everhart–Thornley as secondary electrons detector, using an acceleration voltage of 20 kV and a beam current of 0.1 nA (Catalan Institute of Nanoscience and Nanotechnology, ICN2, Electron Microscopy Unit, Spain). The GO sample was deposited on an Ultrathin Lacey C grid; any excess of material was removed and dried overnight at room temperature. The lateral dimension distribution was obtained by measuring the flakes using ImageJ software. Finally, optical images were acquired with a Nikon Eclipse LV100 microscope in transmittance mode at a magnification of 50×. Only micrometer‐sized flakes were visible under the optical microscope, which resulted in the recording of images for LGO, but no flakes were detectable for USGO (i.e., dimensions below the light diffraction limit). The LGO flakes were measured using ImageJ software. Further characterization techniques applied to these GO materials are detailed in the next section.

### MWCNT Materials

MWCNTs (Mitsui, Japan, type Mitsui‐7), kindly provided by Prof. U. Vogel (National Research Centre for the Working Environment), were heated overnight in the oven at 160–180 °C for depyrogenation. Characteristics of these materials were published before.^[^ ^32^, ^33^, ^34^ ^]^ MWCNTs were then dispersed in water for injection (Gibco, ThermoFisherScientific) containing 0.5% bovine serum albumin (BSA; Gibco, ThermoFisherScientific) and submitted to sonication in a water bath for 5–7 min at 80 W (VWR essential, UK).

### Endotoxin Assay

The potential contamination by endotoxins was evaluated according to Mukherjee et al.^[^ ^78^ ^]^ using mouse primary bone marrow derived macrophages, and all the materials (USGO, LGO, and MWCNT) were tested negative (Figure S12, Supporting Information).

### Absorption Spectroscopy

Data were obtained using a Nanodrop 2000c spectrophotometer (ThermoFisherScientific), and a Hellma QS Quartz micro cuvette. GO samples were prepared in water (concentration range of 2.5–20 µg mL^−1^) and measured at room temperature.

### Fluorescence Emission Spectroscopy

Fluorescence emission was assessed for GO samples at concentration range of 25–200 µg mL^−1^ using a LS‐50B Perkin Elmer Spectrofluorometer, at the excitation wavelength of 525 nm, with both excitation and emission slits set at 20.

### Raman Spectroscopy

Raman spectra were acquired with a confocal Raman microscope (WITec) at room temperature, using a 632 nm laser excitation and grating of 600 g nm^−1^. Single Raman spectra were collected on several spots after irradiation with a power of 1 mW for 10 s and using a 50× objective. Samples were prepared by drop casting 20 µL onto glass coverslip left to dry overnight. The data were analyzed with Origin software (OriginLab Corporation; https://www.originlab.com/). The ratio of D and G band intensities (*I*_D_/*I*_G_) was calculated without baseline correction (D: peak height intensity at ≈1340 cm^−1^; G: at ≈1580 cm^−1^).

### X‐Ray Diffraction Spectroscopy

Data were acquired at the ICN2 X‐Ray Diffraction Facility with X'Pert MPD (Multipurpose diffractometer) equipped with a ceramic X‐ray tube with Cu Κ*α* anode (*λ* = 1.540 Å) as X‐ray source and x'Celerator solid‐state detector in the 2*θ* scan range from 5° to 40°, by drop casting the GO samples.

### Zeta Potential

This was measured using a Zeta‐sizer Nano ZS (Malvern Panalytical Ltd) equipped with disposable capillary cells at the ICN2 Molecular Spectroscopy and Optical Microscopy Facility. Water dispersant settings for refractive index and viscosity, and automatic analysis were used for all GO measurements (20 µg mL^−1^). Each sample was measured three times at room temperature.

### Thermogravimetric Analysis

This was used to determine the weight loss of GO samples using a Pyris 6, Perkin‐Elmer Ltd. A GO sample of 1–2 mg weighed into a ceramic crucible was analyzed from 25 to 995 °C at 10 °C min^−1^ with a nitrogen flow of 20 mL min^−1^.

### X‐Ray Photoemission Spectroscopy

Data were obtained using a Phoibos 150 (SPECS, GmbH) electron spectrometer equipped with a hemispherical analyzer, operating under ultrahigh‐vacuum conditions, and with an Al K*α* (*hν* = 1486.74 eV) X‐ray source, at the ICN2 Photoemission Spectroscopy Facility. Samples were prepared by deposition of 20 µg of GO materials onto 5 × 5 silicon wafers (Ted Pella) left to dry overnight. In order to estimate the photoelectron peak intensities, the CasaXPS software was used (Casa Software Ltd; http://www.casaxps.com).

### Animal Exposure

C57BL/6J female mice (6 to 8 weeks old) were purchased from Envigo, UK. The experiment was randomized and mice were kept in groups of four in ventilated cages with ad libitum access to food and water under controlled environmental conditions (humidity, temperature, and light controlled environment). All procedures were conducted after ethical approval from the UK Home Office, under the Project License no. P089E2E0A. After at least a week of acclimatization, each animal was exposed by oropharyngeal aspiration every 14 days to either 1 µg (low dose) or 10 µg (high dose), to achieve a total cumulative dose of either 3 µg (low dose) or 30 µg (high dose) per animal after three exposures. Briefly, animals were anaesthetized by inhalation of 4% isoflurane in 100% oxygen and then held on a slanted board in order to deliver 30 µL of materials or vehicle (water for injection, Gibco, ThermoFisherScientific). The animals (6 animals per group) were kept for 1, 7 (=1 week), 28 (=4 weeks/1 month), or 84 (=12 weeks/3 months) days after the last exposure.

### Sample Collection

At days 1, 7, 28, and 84 after the last exposure, animals were euthanized by IP injection of pentobarbitone (100 µL). During the autopsy, left lungs were clamped and right lungs were washed with PBS (Merck‐Sigma) to collect BAL fluid (*n* = 6). After lavage, right lungs were cut in small portions and then stored in tubes containing either 1 mL of RNAlater (*n* = 6) (Merck‐Sigma) or 0.5 mL of RIPA Buffer (*n* = 6) (Merck‐Sigma) for RNA or protein extraction respectively. Non‐washed left lungs were collected in tubes containing 1 mL of RPMI medium (Merck‐Sigma) and kept at 4 °C for flow cytometry (*n* = 3) or inflated and kept in formalin (Merck‐Sigma) for histology (*n* = 3).

### BAL Fluid Analysis

BAL fluids were centrifuged at 1500 rpm for 5 min at room temperature (Hettich GmbH). Supernatants were aliquoted and stored at −80 °C (Brunswick, Eppendorf). Pellets were resuspended in PBS (Merck‐Sigma), cells were counted and cytospun at 600 rpm for 5 min with ≈100 000 cells per slide (Hettich GmbH). Slides were then fixed in 100% ice‐cold methanol for 10 min, air‐dried and stored at −20 °C. Differential cell staining was performed using Kwik‐Diff (Shandon, ThermoFisherScientific) following the provider's instructions. The number of neutrophils, eosinophils, mono‐ and multinucleated macrophages, and lymphocytes were assessed using optical microscopy (AxioOberver, Zeiss). Colorimetric bright‐field images (Figure 5c; Figure S1a, Supporting Information) were generated with a slide scanner (Pannoramic 250 Flash, 3DHistech Ltd). The protein content in BAL supernatants was evaluated using a BCA assay (Pierce BCA Protein Assay Kit, ThermoFisherScientific) following provider's instructions. Absorbance values were recorded at 562 nm using a spectrophotometer (Varian Cary 50, Agilent) and concentration values were calculated using a standard curve.

### Cell Phenotyping Using Flow Cytometry

Harvested lungs were cut into small parts and digested using 0.4 Wünsch units mL^−1^ of Liberase (Roche) and 0.01 mg mL^−1^ of DNase I (Roche) in RPMI medium (Merck‐Sigma). After shaking for 30 min at 37 °C (MaxQ, ThermoFisherScientific), 5% fetal bovine serum (FBS; Gibco, ThermoFisherScientific) was added and the digested lungs were filtered through 100 µm filter meshes (Greiner Bio‐one). RPMI medium containing 5% of FBS (Gibco, ThermoFisherScientific) and 5 mm of EDTA (Gibco, ThermoFisherScientific) was added to prevent cell aggregation. Cells were centrifuged for 5 min at 2300 rpm, supernatants were discarded, and red blood cells were lysed for 7 min using 500 µL of Red Blood Cell Lysing Buffer (Hybri‐Max, Merck‐Sigma Aldrich). After centrifugation at 2300 rpm for 5 min (Hettich GmbH), cell pellets were suspended in 1 mL of RPMI medium (Merck‐Sigma). Cells were counted using a haemocytometer and incubated using a Live/Dead UV viability dye (ThermoFisherScientific) for 20 min at room temperature for cell death evaluation. After a washing step (centrifugation at 2300 rpm for 5 min and washing with PBS), cells were suspended in 100 µL of staining buffer (3% FBS and 0.05% sodium azide (Merck‐Sigma) in PBS). Non‐specific sites were blocked with anti‐mouse CD16/CD32 (Fc block, eBiosciences) for 5 min and cells were incubated with fluorochrome‐conjugated antibodies (see Table S4, Supporting Information) for 30 min at 4 °C for staining of specific cell membrane receptors. Compensations, fluorescence minus one and controls were also prepared. After washing with PBS, cells were finally fixed at room temperature for 20 min with 4% of PFA (Merck‐Sigma) in PBS, washed again with PBS, and kept overnight at 4 °C in staining buffer (3% FBS and 0.05% sodium azide in PBS). The next day, cells were washed with PBS and suspended in 100 µL of permeabilization buffer (0.5% tween 20 with 1% FBS and sodium azide 0.01 in PBS). After incubation for 30 min at 4 °C and a PBS washing step, 5% FBS in PBS was added for 20 min to block non‐specific intracellular binding sites. Cells were washed with PBS, suspended in 100 µL of permeabilization buffer, and then stained for 60 min at room temperature in the dark for intracellular staining with Arginase (Table S4, Supporting Information). After a final PBS washing step, cells were suspended in 300 µL of staining buffer and analyzed by flow cytometry using a BD LSR II (BD Biosciences), for evaluating variation of immune cell populations in the whole lung. Granulocyte, macrophage, monocyte, dendritic cells, and lymphocyte populations and their subsets were isolated using specific gating (see Figure S13, Supporting Information).

### Cytokine Levels by ELISA

Harvested lungs were digested in 1 mL of RIPA buffer (Merck‐Sigma) supplemented with EDTA‐free protease inhibitor (complete Mini, Roche), and homogenized with 5 mm stainless steel beads using a TissueLyser LT system (Qiagen), operating at 50 Hz for 10 min. Cell lysates were then centrifuged for 5 min at 2600 g (Hettich GmbH), and supernatants were collected and stored at −80 °C until analysis. After measurement of total protein contents using a BCA assay (Pierce, ThermoFisherScientific), the concentrations of the cytokines IL‐1*α*, IL‐1b, IL‐6, TNF‐*α*, MCP‐1, GM‐CSF, IL‐17A, IL‐23, IL‐12p70, IFN‐*γ*, IFN‐*β*, IL‐27, and IL‐10 were evaluated using the multiplex Mouse Inflammation Panel (13‐plex, v‐plate, Biolegend) according to manufacturer's instructions. Fluorescence intensities were measured using a BD FACSVerse flow cytometer (BD Biosciences) and concentration values were extrapolated from standard curves. IgE and IL‐4 concentrations were evaluated using single ELISA kits (Biolegend). For each sample, cytokine concentrations were normalized by the total protein content and were expressed in pg mg^−1^ of protein.

### Gene Expression by Multiplex RT‐qPCR

Prior to RT‐qPCR analysis, lung samples were kept in RNAlater storage solution (Merck‐Sigma) until processing. After removing of RNAlater and washing with PBS, lungs were lysed in 1 mL of lysis buffer containing 2‐mercaptoethanol (Gibco, ThermoFisherScientific). After homogenization using 5 mm stainless steel beads and a TissueLyser LT (Qiagen) running at 50 Hz for 10 min, lysed lungs were centrifuged at 2600 g for 5 min (Hettich GmbH) to remove cell debris. Supernatants were kept at −80 °C prior to extraction. Total RNA was extracted using spin cartridges containing silica membranes (PureLink RNA Mini kit, Qiagen) according to the manufacturer's instructions. Briefly, after addition of 1 volume of 70% ethanol (ThermoFisherScientific) for 1 volume of sample to precipitate RNA, samples were loaded in the cartridge. After washing steps and addition of DNase, the extracted RNA was eluted using 30 µL of RNase‐Free Water (Gibco, ThermoFisherScientific). Total RNA concentration was measured and purity was calculated using a Biophotometer Plus spectrophotometer (Eppendorf AG). RNA samples were kept at −80 °C until processing. For each sample, first‐strand cDNA was synthesized from 1 µg of extracted RNA using the High Capacity cDNA Reverse Transcription kit (Applied Biosystems). Briefly, 10 µL of reverse transcription Master Mix and 10 µL of sample were incubated for 10 min at 25 °C, followed by 2 h at 37 °C, and 5 min at 85 °C in a CFX96 real‐time PCR system (BioRad). The expression of pre‐selected 48 genes (Table S5, Supporting Information) was evaluated in lung samples by Multiplex RT‐qPCR in a Biomark HD system (BioMark HD 96.96 IFC, Fluidigm) using extracted cDNA (30 µl at 1 ng µL^−1^) and primers (10 µL at 50 µm) (Table S5, Supporting Information), according to the manufacturer's instructions. Samples were analyzed in duplicates. For every sample, ΔΔ*Ct* values were calculated, normalized using house‐keeping genes, and data were finally expressed in fold change compared to the negative control.

### Histology Block Preparation

After sampling, inflated lungs were stored in formalin 10% (Merck‐Sigma) for 24 h and then transferred to vials containing ethanol 70% (ThermoFisherScientific). The lungs were embedded in paraffin, and sections of 5.0 µm of thickness were obtained using a microtome (RM2255, Leica Biosystems).

### Histological Staining

For histopathological analysis, sections were stained with hematoxylin and eosin using an automatic stainer (XL autostainer, Leica Biosystems). Bright‐field images were generated with a slide scanner (Pannoramic 250 Flash, 3DHistech Ltd). Lung perimeter, pleural and bronchial thickness, and granulomatous and BALT areas of each sample were identified and analyzed using CaseViewer (software version 2.4.0.11902, 3DHISTECH Ltd). Masson's Trichrome staining of lung sections was also performed for the 28 and 84 days samples only. Level of collagen deposition was determined by automatic pixel quantification of five images (region of interest) for each section using Python software (v. 3.9.1).

### TUNEL Assay

Quantitation of apoptotic cells in the paraffin embedded lung sections was performed using the DeadEnd Fluorometric TUNEL System (Promega). As pre‐treatment, sections were dewaxed and rehydrated in series of ethanol solutions and water. For detection, sections were then fixed in paraformaldehyde (PFA 4% in deionized water; Merck‐Sigma) and permeabilized with Proteinase K solution (20 µg mL^−1^; Merck‐Sigma) for 10 min. Next, sections were fixed again in PFA 4% and left in the equilibration buffer for 10 min. After equilibration, sections were incubated with 50 µL of the TdT reaction mix, covered with plastic coverslips, and placed in a humidified chamber at 37 °C away from light for 60 min. After reaction, sections were washed with the Stop Reaction buffer, PBS, then stained with DAPI (Merck‐Sigma), and mounted with coverslips using mounting media. Imaging was performed with an epifluorescence microscope (Axio Oberver, Zeiss) and the corresponding filter for detecting fluorescein emission. The quantification of apoptosis was calculated based on the number of positive cells relative to the negative control.

### Immunohistochemistry

Lung tissue remodeling and fibrosis were evaluated in lung sections by immunohistochemical quantification of collagen I (rabbit anti‐mouse collagen‐I, #34710, Abcam). Briefly, the lung sections were first dewaxed with Histo‐clear (HistoChoice clearing agent, Merck‐Sigma), followed by rehydration in series of ethanol solutions and water. Antigen retrieval was performed with citric acid bath (pH = 6) and microwave heating for 10 min. After cooling down at room temperature, the sections were permeabilized with 0.01% Triton X‐100 (Merck‐Sigma) in PBS (Merck‐Sigma) for 10 min. Blocking was performed with heat‐inactivated normal donkey serum (10%, Merck‐Sigma) in 1% BSA (Gibco, ThermoFisherScientific) solution for 2 h. The sections were then gently drained with a pipette to remove the excess blocking buffer, and primary antibodies in PBS solution (Merck‐Sigma) with 1% BSA (Merck‐Sigma) were applied; sections were incubated overnight at 4 °C. The following day, sections were washed with 0.01% Triton X‐100 in PBS and then incubated at room temperature for 1 h with secondary antibodies (donkey anti‐rabbit Alexa fluor 555, #31572, ThermoFisherScientific) suspended in PBS solution with 1% BSA, away from direct light. After incubation, sections were gently washed in water, dried, and then mounted with mounting medium (ProLong Gold Antifade Mountant with DAPI, Molecular Probes, ThermoFisherScientific) and glass coverslips. The fluorescence intensity of each section was measured by epifluorescence microscopy (Axio Oberver, Zeiss), and semi‐quantify using ImageJ software (US National Institutes of Health, Bethesda).

### Evaluation of Clearance Using Raman Spectroscopy Imaging

Deparaffinized lung sections and cytospun BAL cells fixed with ice‐cold methanol were scanned by Raman microscopy (XploRA Plus, HORIBA) using a laser excitation wavelength of 638 nm and a grating of 600, with 3 µm of distance between each point. GO was identified based on characteristic D (≈1340 cm^−1^) and G (≈1580 cm^−1^) bands (Figure S14, Supporting Information). Only G bands were used to evaluate the interaction/internalization because less interferences were observed with biological tissues around 1580 cm^−1^. The internalization of GO sheets in neutrophils and macrophages was evaluated by overlapping the maps of GO intensities with the BAL cell bright‐field images (the authors counted 30 to 100 neutrophils and around 150 macrophages per sample; *n* = 3). Only interaction of GO with neutrophils and macrophages was reported, as the authors did not observe GO sheets interacting with the other cells present in BAL fluids (i.e., eosinophils, lymphocytes). The number of GO‐positive cells (interacting with GO) and GO‐negative cells (not interacting with GO) was recorded. For each GO‐positive cell, the relative quantity of material internalized was evaluated based on the number of GO‐positive pixels counted, divided by the total cell surface area (in pixel), and expressed as average loading in GO‐positive cells (%). The total loading (%) per cell type was then calculated by multiplying the percentage of GO‐positive cells by the average loading in GO‐positive cells (%).

### Statistical Analysis

Data are expressed as mean ± standard deviation. Statistical analysis was performed using Graphpad Prism 8.0 (GraphPad Software Inc., San Diego, CA). For flow cytometry and TUNEL, two‐way ANOVA followed by Dunnett's post‐hoc test was used to evaluate statistical differences compared to the control (*n* = 3; ^*^ *p* < 0.05, ^**^ *p* < 0.01, and ^***^ *p* < 0.001). Two‐way ANOVA followed by Dunnett's post‐hoc test was also used for the histopathological analysis to evaluate differences compared to the negative control or differences between different time‐points (*n* = 3; *p* < 0.05:^*^, *p* < 0.01:^**^, *p* < 0.001:^***^). For BALF, gene expression, protein concentration, and collagen deposition via immunostaining or Masson Trichrome, one‐way ANOVA followed by Dunnett's post‐hoc test or Kruskall–Wallis followed by Dunn's post‐hoc test, depending on data normality, was used to evaluate significant differences compared to the negative control at each time‐point (*n* = 4–6; *p* < 0.05:^*^, *p* < 0.01:^**^, *p* < 0.001:^***^). For Raman analysis, at each time‐point, *t*‐test or Man–Whitney test, depending on data normality, were used to evaluate statistical differences between the LGO and USGO (*n* = 3; *p* < 0.05:^*^, *p* < 0.01:^**^, *p* < 0.001:^***^). As responses to MWCNT exposure were extremely positive for most endpoints tested, an additional comparison between LGO, USGO, MWCNTs, and the negative control was performed separately to avoid false negative results.

## Conflict of Interest

The authors declare no conflict of interest.

## Author Contributions

T.L. and L.A.V.L. contributed equally to this work. T.L.: Conceptualization (lead); investigation (lead for biological analyses); formal analysis (lead); methodology (lead); visualization (lead); writing—original draft (lead); writing—review and editing (equal). L.A.V.L.: Conceptualization (lead); investigation (lead for biological analyses); formal analysis (lead); methodology (lead); visualization (lead); writing—original draft (lead); writing—review and editing (equal). A.F.: Investigation (supporting); visualization (supporting); writing—review and editing (equal). A.A.: Investigation (supporting). K.B.: Methodology (lead); software (lead). N.L.: Investigation (lead for materials); resources (lead for GO materials); formal analysis (lead for GO materials); visualization (lead for materials); supervision (supporting); writing—review and editing (equal). K.K.: Conceptualization (lead); resources (lead for GO materials); funding acquisition (lead); supervision (lead); writing—review and editing (equal). C.B.: Conceptualization (lead); investigation (supporting); resources (lead for MWCNT materials); funding acquisition (lead); supervision (lead); visualization (supporting); writing—review and editing (equal).

## Supporting information

Supporting Information

## Data Availability

The data that support the findings of this study are available from the corresponding author upon reasonable request.
