# Supplementary material for: Innate but Not Adaptive Immunity Regulates Lung Recovery from Chronic Exposure to Graphene Oxide Nanosheets
Source: Adv Sci (Weinh). 2022 Feb 15;9(11):2104559. doi: 10.1002/advs.202104559 (PMC9008410; doi:10.1002/advs.202104559)
Supplement: Supplementary file 1 — Supporting Information [file ADVS-9-2104559-s001.pdf]

ADVANCED SCIENCE
Open Access

Supporting Information

for *Adv. Sci.,* DOI: 10.1002/advs.202104559

Innate but not adaptive immunity regulates lung recovery from chronic exposure to graphene oxide nanosheets

Thomas Loret, Luis Augusto Visani de Luna, Alexander Fordham, Atta Arshad, Katharine Barr, Neus Lozano, Kostas Kostarelos, and Cyrill Bussy**

SUPPLEMENTARY INFORMATION

Innate but not adaptive immunity regulates lung recovery from chronic exposure to graphene oxide nanosheets

Thomas Loret[1,2,3]^, Luis Augusto Visani de Luna[1,2,3]^, Alexander Fordham[1,2,3], Atta Arshad[1,2,3], Katharine Barr[1,2], Neus Lozano[4], Kostas Kostarelos[1,2,4,]* and Cyrill Bussy[1,2,3,]*

SUPPLEMENTARY TABLES

	Technique	USGO	LGO
**Physicochemical properties**			
Lateral dimension	Optical microscopy	Non detectable (< 2μm)	1.0 μm - 25.0 μm<br>95% < 21.0 μm<br>Mean 8.1 μm<br>[n=268]
	SEM	10 nm - 503 nm<br>95% < 250 nm<br>Mean 48 nm<br>[n=636]	2.5μm - 16.5 μm<br>95% < 15.0 μm<br>Mean 8.2 μm<br>[n=14]
	AFM	20 nm - 790 nm<br>95% < 210 nm<br>Mean 59 nm<br>[n=4268]	3.0 μm - 17.0 μm<br>[n=14]
Thickness	AFM	1 - 2 nm	1 - 2 nm
Optical properties	Absorption spectroscopy	ε_{230} (mL μg^{-1} cm^{-1})= 0.041	ε_{230} (mL μg^{-1} cm^{-1})= 0.040
	Fluorescence λ_{600} (λexc_{525})	0.915 * C_{GO} (μg/mL)	0.977 * C_{GO} (μg/mL)
Degree of defects (I_D/I_G)	Raman spectroscopy	1.18 ± 0.03	1.20 ± 0.02
Peak (2θ)<br>Interlayer distance (nm)	XRD	11.53 °<br>0.76	12.31 °<br>0.71
Surface charge (ζ-Potential)	Electrophoretic mobility	-53.5 ± 2.2 mV	-49.0 ± 0.9 mV
Functionalization degree	TGA	30-75°C: 7% (water)<br>200-250°C: 23%<br>250-950°C: 13%<br>TOTAL 36%	30-75°C: 8% (water)<br>200-250°C: 23%<br>250-950°C: 15%<br>TOTAL 38%
Chemical composition	XPS	C: 73.0%, O: 24.8%, N: 0.4%, S: 1.4%, B: 0.4%	C: 70.7%, O: 26.2%, N: 1.4%, S: 1.2%, B: 0.5%
Purity (%C + %O)		97.8%	96.9%
C:O ratio		2.9	2.7
π-π^*,O-C=O, C=O, C-O, C=C		0.2%, 5.8%, 7.5%, 37.3%, 49.1%	0.9%, 3.8%, 5.3%, 46.1%, 43.9%

Table S1. Summary of the physicochemical characterization of USGO and LGO sheets.

Groups	Days	Granulomatous Area (%)		BALT Area (%)	
		Low dose	High dose	Low dose	High dose
Control	1	-	-	-	-
	7	-	-	-	-
	28	-	-	-	-
	84	ND	-	ND	-
LGO	1	-	1.6 ± 2.0	-	0.06 ± 0.11
	7	-	0.44 ± 0.37	-	0.26 ± 0.39
	28	-	0.12 ± 0.06	-	-
	84	ND	0.07 ± 0.03	ND	-
USGO	1	-	0.12 ± 0.11	-	0.24 ± 0.28
	7	-	0.04 ± 0.03	-	0.52 ± 0.78
	28	-	-	-	-
	84	ND	0.01 ± 0.01	ND	-
MWCNT	1	1.24 ± 0.14	2.6 ± 2.53	2.88 ± 1.04	3.4 ± 0.96
	7	0.15 ± 0.02	10.6 ± 0.15	1.46 ± 0.98	5.78 ± 1.33
	28	2.77 ± 0.95	2.21 ± 3.43	0.16 ± 0.01	0.18 ± 0.26
	84	ND	1.44 ± 0.78	ND	0.17 ± 0.04

Mean value and standard deviation (n=3). ND: Non determined.

Table S2. Histopathology analysis for transient immune structures in lungs.

Groups	Days	Pleural thickness (μm)		Bronchial thickness (μm)	
		Low dose	High dose	Low dose	High dose
Control	1	6.8 ± 1.02	8.31 ± 0.61	27.8 ± 2.28	29.89 ± 2.0
	7	5.8 ± 0.17	6.51 ± 0.81	28.8 ± 2.62	28.02 ± 3.2
	28	6.2 ± 0.76	7.87 ± 0.24	28.1 ± 0.59	22.61 ± 1.6
	84	ND	9.15 ± 0.81	ND	26.05 ± 1.4
USGO	1	6.5 ± 1.51	8.47 ± 0.19	28.7 ± 1.90	33.38 ± 4.5
	7	6.1 ± 0.94	7.09 ± 2.24	28.4 ± 0.81	31.26 ± 1.09
	28	5.9 ± 0.73	9.17 ± 0.13	25.8 ± 1.05	26.45 ± 2.57
	84	ND	8.38 ± 1.1	ND	28.17 ± 3.46
LGO	1	6.5 ± 1.66	9.64 ± 1.68	29.3 ± 2.26	29.76 ± 2.10
	7	5.8 ± 0.63	7.53 ± 1.0	26.0 ± 2.09	29.57 ± 1.61
	28	6.1 ± 1.41	10.77 ± 2.31	26.9 ± 0.14	24.36 ± 5.29
	84	-	9.17 ± 2.28	-	25.14 ± 2.50
MWCNT	1	14.5 ± 4.92*	13.3 ± 2.73	39.2 ± 0.03*	40.60 ± 10.93*
	7	7.0 ± 1.06#	8.44 ± 1.58	36.9 ± 0.42*	39.45 ± 8.35*
	28	5.0 ± 0.81#	12.88 ± 3.23*	28.1 ± 4.22#	31.89 ± 1.50*
	84	ND	15.52 ± 5.77*	ND	34.70 ± 6.41

Mean value and standard deviation (n=3). Statistical comparison to Control at same time-points: (*) P <0.05. Statistical comparison through time for each material: (#) P <0.05 showing significance compared to day 1. ND: Non determined.

Table S3. Histopathology analysis for morphological changes in lungs.

Antigen	Fluorophore	Clone	Supplier	Dilution
CD45R/B220	Pacific blue	RA3-6B2	BioLegend	1:100
Arginase-1	eF450	A1exF5	eBioscience	1:200
CD38	APC-cy7	90	BioLegend	1:200
Ly6G	FITC	1A8	BioLegend	1:100
CD11b	PE-Cy7	M1/70	BioLegend	1:200
Ly6C	BV605	HK1.4	eBioscience	1:200
CD11c	BV650	N418	BioLegend	1:200
CD45	BV510	30-F11	BioLegend	1:400
CD3	BV711	17A2	BioLegend	1:200
CD4	APC	GK1.5	BioLegend	1:200
CD8	PerCP-Cy5.5	53-6.7	BioLegend	1:200
CD64	PE	X54-5/7.1	BioLegend	1:200
Siglec F	PE-CF594	E50-2440	BD Bioscience	1:200
MHC II	BV785	M5/114.15.2	BioLegend	1:500
Fc Block (CD16/CD32)		93	eBioscience	1:100
Live/Dead	UV		Life Technologies	1:2000

Table S4. List of antibodies used for flow cytometry.

Gene	Forward sequence (5'-3')	Reverse sequence (5'-3')
IL-6	ATGGATGCTACCAAACTGGA	CCTCTTGGTTGAAGATATGA
TNF-a	CAGACCCTCACACTCAGATCATCT	CCTCCACTTGGTGGTTTGCTA
IL-10	GGTTGCCAAGCCTTATCGGA	ACCTGCTCCACTGCCTTGCT
Spp1	CTGGCAGCTCAGAGGAGAAG	ACAGGGATGACATCGAGGGA
IL-4	GAGACTCTTTCGGGCTTTTC	TGATGCTCTTTAGGCTTTCCA
Arginase-1	CTTGCGAGACGTAGACCCTG	TGAGTTCCGAAGCAAGCCAA
IL-9	AATGCCACCTCTCAGCCTTC	AGAATGCCCACATCCCTTGG
SAA-3	AACTATGATGCTGCCCGGAG	GCTCCATGTCCCGTGAACTT
CXCL1	TGGCTGGGATTCACCTCAAG	CCGTTACTTGGGGACACCTT
IL-5	AGGCTTCCTGTCCCTACTCA	CCCCCACGGACAGTTTGATT
IL-33	CTCACTGCAGGAAAGTACAGCA	TATTTTGCAAGGCGGGACCA
IL-2	TGTGCTCCTTGTCAACAGCG	GTGAGCATCCTGGGGAGTTT
HSP70	TTGGGCACCGATTACTGTCA	CCAGGCTACTGGAACACTGA
Colagen-1	CGATGGATTCCCGTTCGAGT	GCTGTAGGTGAAGCGACTGT
Casp9	CTAGTGAGCGAGCTGCAAGT	CCAGATCCTGCCTGCTGAATA
Casp8	CCTCCTCCTATGTCCTGTCTCA	TGCCTGAGCTACCTGGAGAGT
Casp1	ACTGCTATGGACAAGGCACG	GCAAGACGTGTACGAGTGGT
Casp2	GACTGCCTACTCGCTCAGAC	ACATCTCCTTGCATCGGTGG
Casp3	GAGCTTGGAACGGTACGCTA	CCGTACCAGAGCGAGATGAC
MMP-9	CCGACTTTTGTGGTCTTCCCC	ATGTCTCGCGGCAAGTCTTC
MMP-3	CCCTGCAACCGTGAAGAAGA	GACAGCATCCACCCTTGAGT
MMP-2	AACGGTCGGGAATACAGCAG	AAACAAGGCTTCATGGGGGC
GSH	GCCTGAGTCTAAAGGTGGTGA	TGACCTTGTCCCCTGCAAAC
HO-1	CATAGCCCGGAGCCTGAATC	CTCAGCATTCTCGGCTTGGA
p53	TCAAGGAGGAAAGCCCAAACT	CAGACAGGCTTTGCAGAATGGAA
NRF2	AACAGAACGGCCCTAAAGCA	TGGGATTCACGCATAGGAGC
CYP1A1	CCCACAGCACCACAAGAGATA	AAGTAGGAGGCAGGCACAATGTC
CYP1B1	ACATCCCCAAGAATACGGTC	TAGACAGGTTCCTCACCGATG
SOD1	ACAAAGACTGGAAATGCTGGGAGC	AGACTCAGACCACACAGGGAATGT
SOD2	AGGCTGTGAATGGAGTTCAGTGGT	TAAGCACTCCTCAAGCAGGAGCAA
Ki67	CTGGTCACCATCAAGCGGAG	CAATACTCCTTCCAAACAGGCAG
aSMA	ACTCTCTTCCAGCCATCTTTCA	ATAGGTGGTTTCGTGGATGC
TGF-b	AACTATTGCTTCAGCTCCACAGAG	GTTGGCATGGTAGCCCTTG
Vimentin	GCGAGAGAAATTGCAGGAGGA	CGTTCAAGGTCAAGACGTGC
GAPDH	AGCCCTTGAGCCTTATTGTC	TGCACTACGCCATAACTACC

Table S5. List of PCR primers.

SUPPLEMENTARY FIGURES LEGENDS

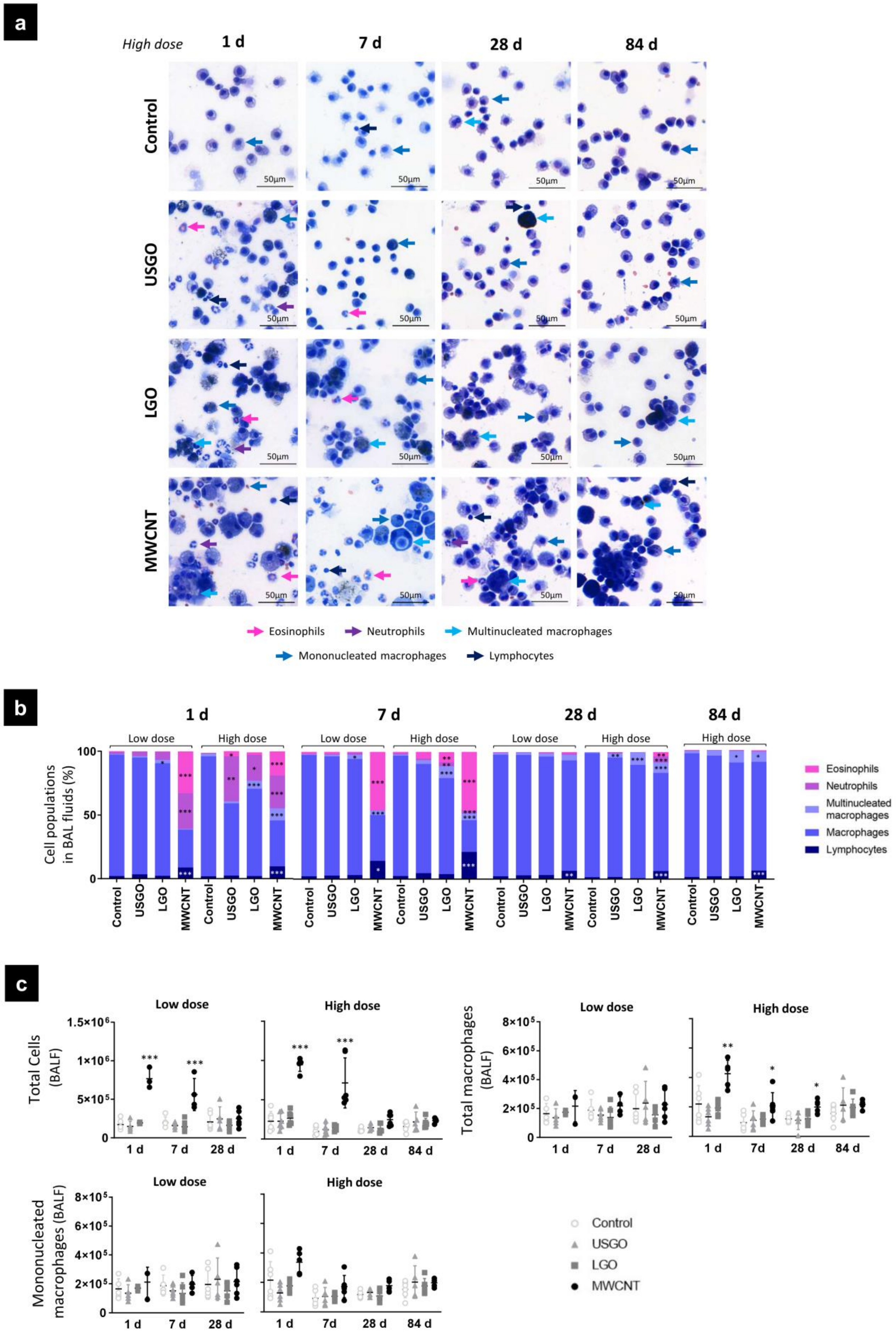
a
High dose
1 d
7 d
28 d
84 d
Control
USGO
LGO
MWCNT
50µm
Eosinophils
Neutrophils
Multinucleated macrophages
Mononucleated macrophages
Lymphocytes
b
1 d
7 d
28 d
84 d
Low dose
High dose
Cell populations in BAL fluids (%)
100
50
0
Control
USGO
LGO
MWCNT
Eosinophils
Neutrophils
Multinucleated macrophages
Macrophages
Lymphocytes
c
Low dose
High dose
Total Cells (BALF)
1.5×10⁶
1×10⁶
5×10⁵
Total macrophages (BALF)
8×10⁵
6×10⁵
4×10⁵
2×10⁵
Mononucleated macrophages (BALF)
1 d
7 d
28 d
84 d
Control
USGO
LGO
MWCNT

Figure S1. Influx of immune cells in the alveolar space. Mice were exposed by oropharyngeal aspiration to three times 1 µg (Low dose) or 10 µg (High dose) of nanomaterials for 28 days. One, 7, 28, 84 days after the last exposure, bronchoalveolar lavages fluids (BALF) were collected from the right lung. Collected cells were stained with colorimetric dyes and counted using optical microscopy. For each time-point, one-way ANOVA followed by Dunnett's post-hoc test was used to evaluate significant differences compared to the negative control (n=6; p<0.05:*, p<0.01:**, p<0.001:***)

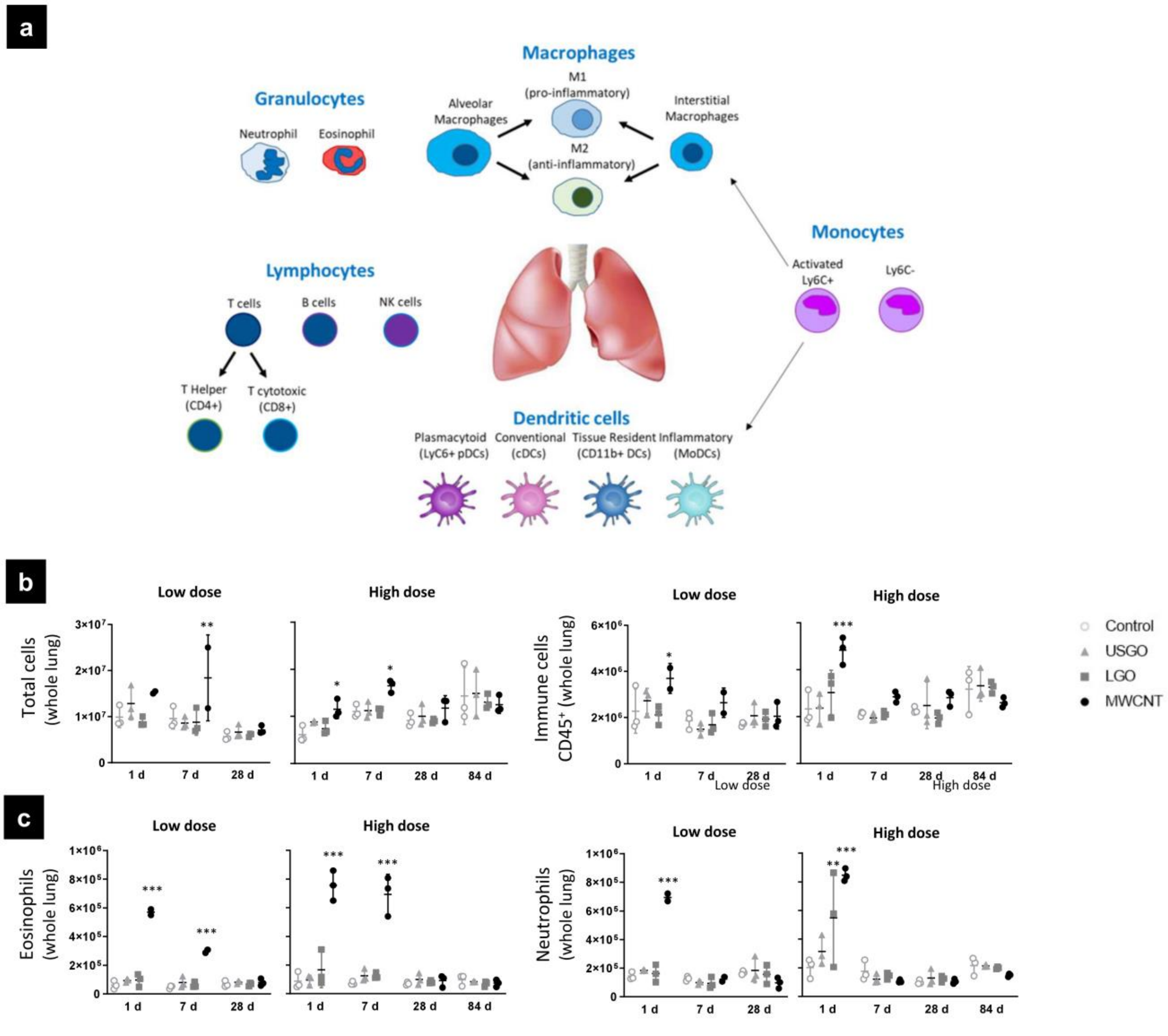

Figure S2. Cells phenotyping strategy, total cells, immune cells and granulocytes number in the whole lung. Mice were exposed by oropharyngeal aspiration to three times 1 µg (Low dose) or 10 µg (High dose) of nanomaterials for 28 days. One, 7, 28, 84 days after the last exposure, left lungs (Whole lung) were digested and collected cells were stained with antibodies for phenotyping of immune cells by flow cytometry. (**a**) Immune cells phenotyped in the whole lung by flow cytometry. (**b**) Total cells and immune cells (CD45+) number. (**c**) Granulocytes number. Neutrophils were identified based on their high expression of Ly6G. Eosinophils were identified based on their high scattering value and high Siglec-F, intermediate CD11b and low MHC II expression. Two-way ANOVA followed by Dunnett's post-hoc test was used to evaluate

changing in the number of cells in the whole lung (n=3; p<0.05:*, p<0.01:**, p<0.001:***).

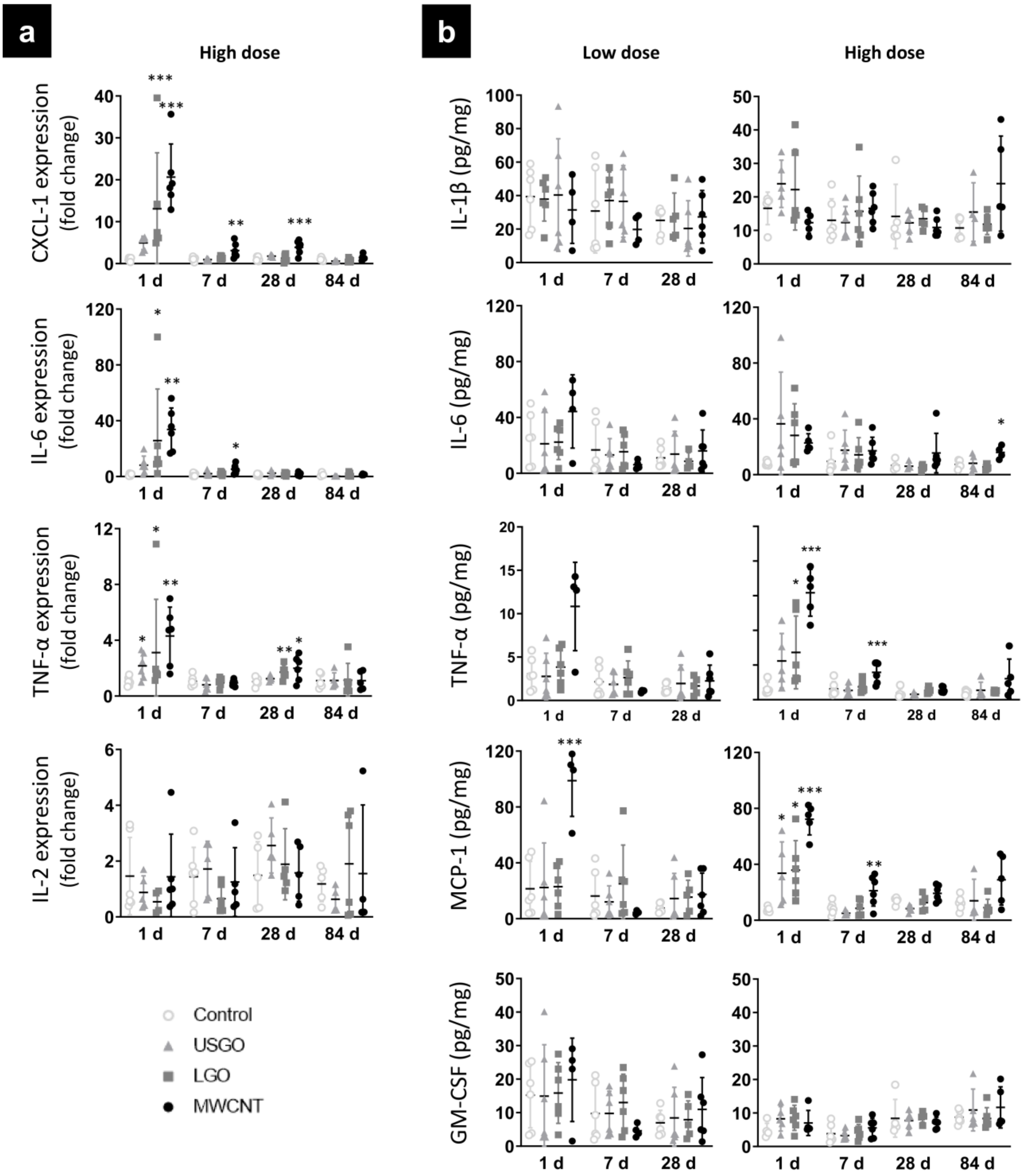

Figure S3. Levels of inflammatory mediators linked to innate immunity. Mice were exposed by oropharyngeal aspiration to three times 1 µg (Low dose) or 10 µg (High dose) of nanomaterials for 28 days. One, 7, 28, 84 days after the last exposure, lungs were collected and then lysed to evaluate differences in gene expression compared to the negative control by RT-qCPR (**a**) or protein concentration by ELISA (**b**). For proteins, each sample was normalized by its own total protein concentration evaluated using a BCA assay. At each time-point, one-way ANOVA followed by Dunnett's post-hoc test or Kruskall-Wallis followed by Dunn's post-hoc test was used to evaluate statistical differences compared to the negative control (n=6; p<0.05:*,

p<0.01:**, p<0.001:***).

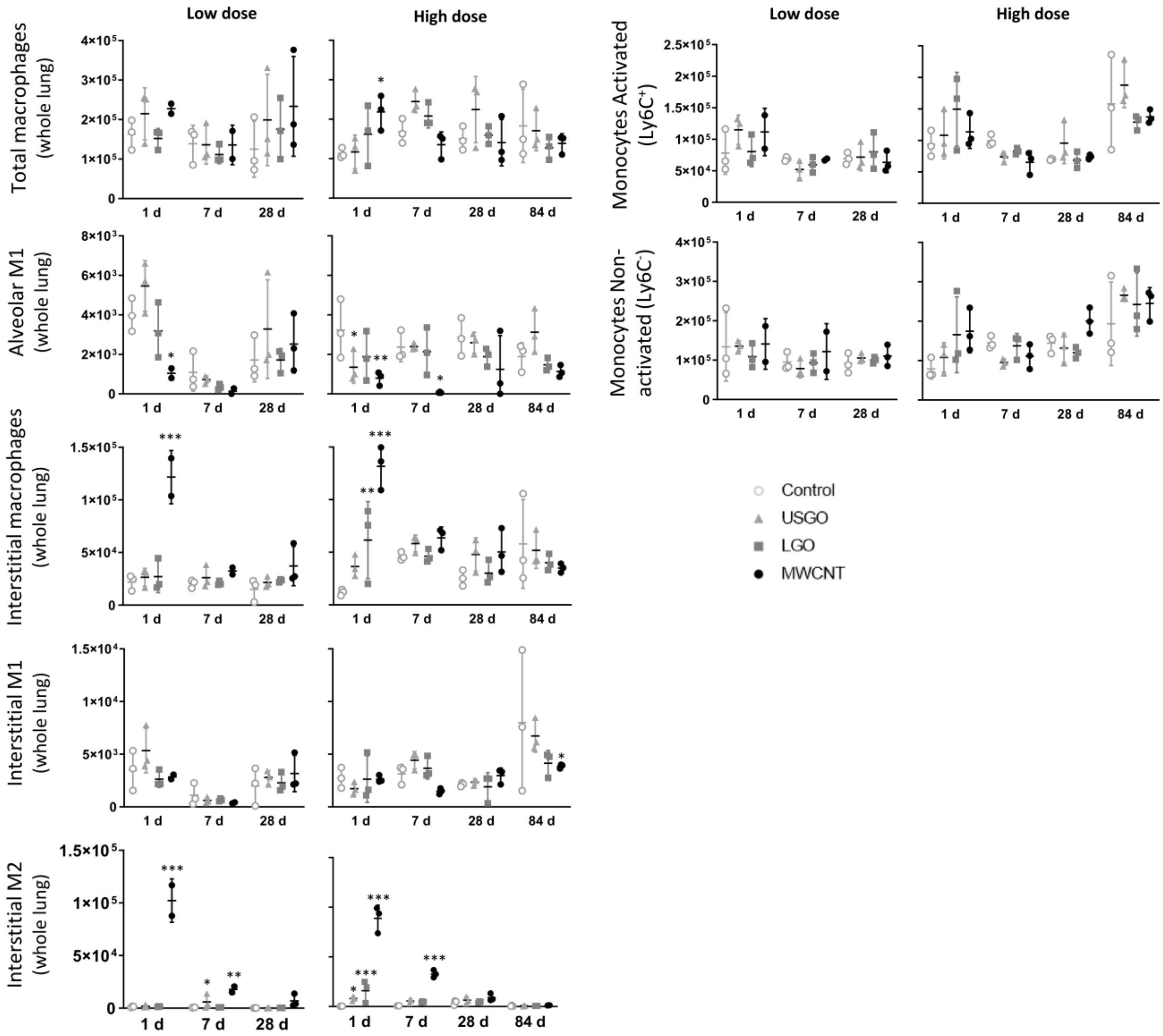

Figure S4. Macrophages and monocytes populations in the whole lung. Mice were exposed by oropharyngeal aspiration to 1 µg (Low dose) or 10 µg (High dose) of nanomaterials, applied three times, over a 28 day period. One, 7, 28, 84 days after the last exposure, left lungs (whole lung w/o lavage) were digested and individualised cells were stained with antibodies for phenotyping of macrophages and monocytes by flow cytometry. Macrophages were isolated based on their high CD64 expression. Alveolar macrophages were confirmed to be $CD11c^+$ and Siglec-F^+, whereas interstitial macrophages were CD11b intermediate/$^+$ and Siglec-F^-. Macrophage activation towards pro-inflammatory (M1) and anti-inflammatory (M2) subsets was evaluated using CD38 (positive for M1) and Arginase-1 (positive for M2). Activated and non-activated monocytes were differentiated according to their expression of Ly6C. Two-way ANOVA followed by Dunnett's post-hoc test was used to evaluate difference in the number of lymphocytes compared to the negative control (n=3; p<0.05:*, p<0.01:**, p<0.001:***).

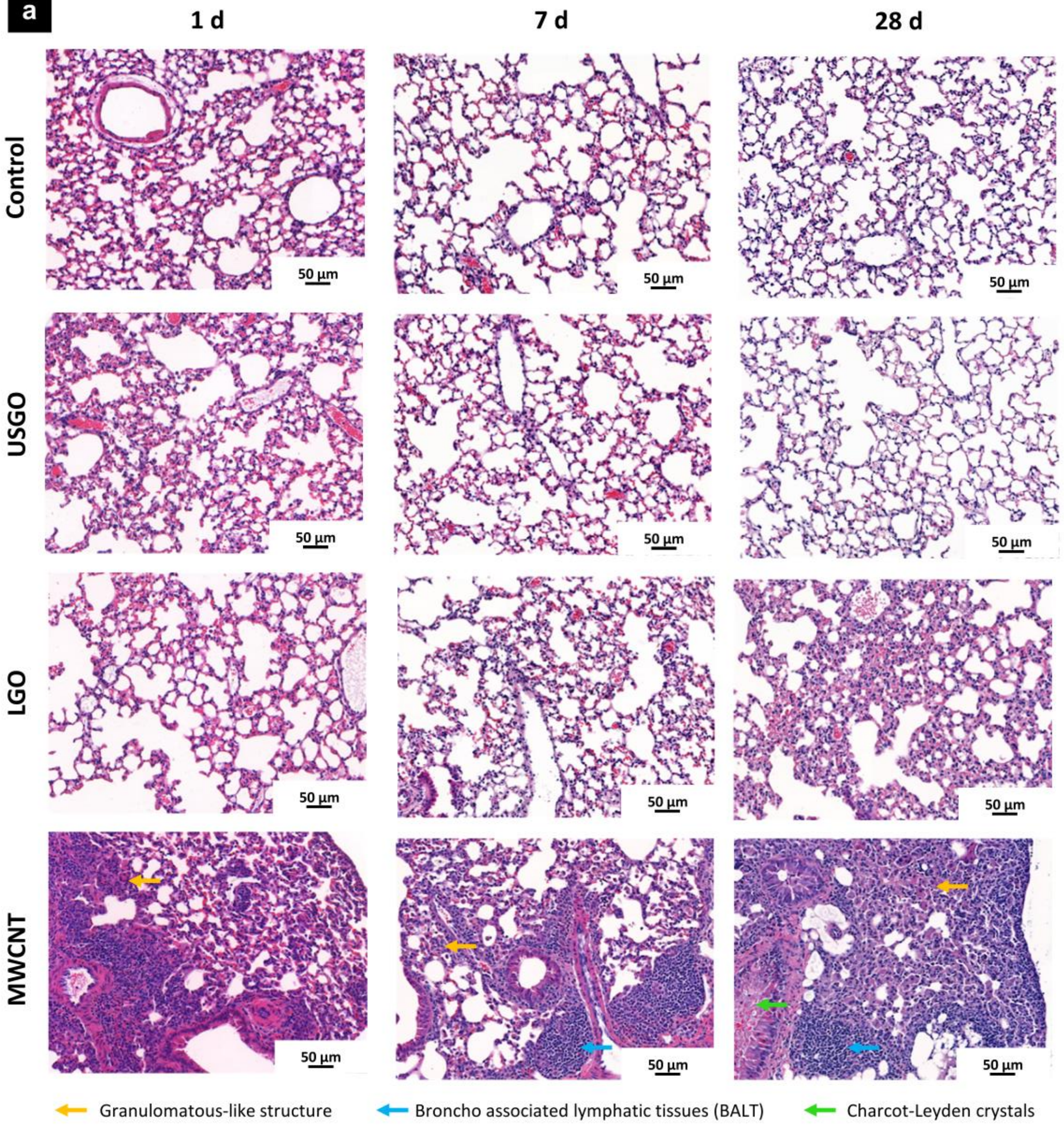
a
1 d
7 d
28 d
Control
USGO
LGO
MWCNT
50 µm
Granulomatous-like structure
Broncho associated lymphatic tissues (BALT)
Charcot-Leyden crystals

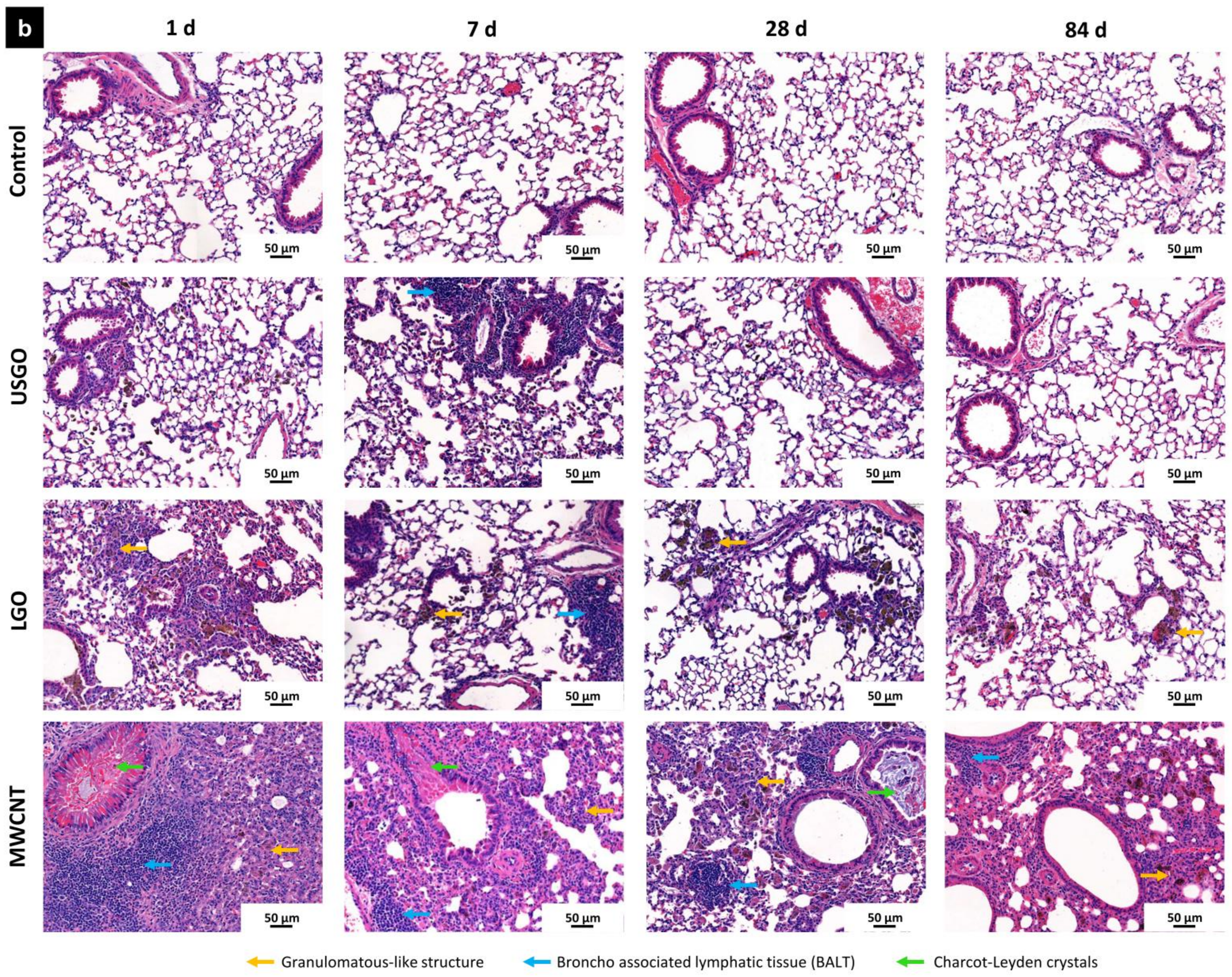
b
1 d
7 d
28 d
84 d
Control
USGO
LGO
MWCNT
50 µm
Granulomatous-like structure
Broncho associated lymphatic tissue (BALT)
Charcot-Leyden crystals

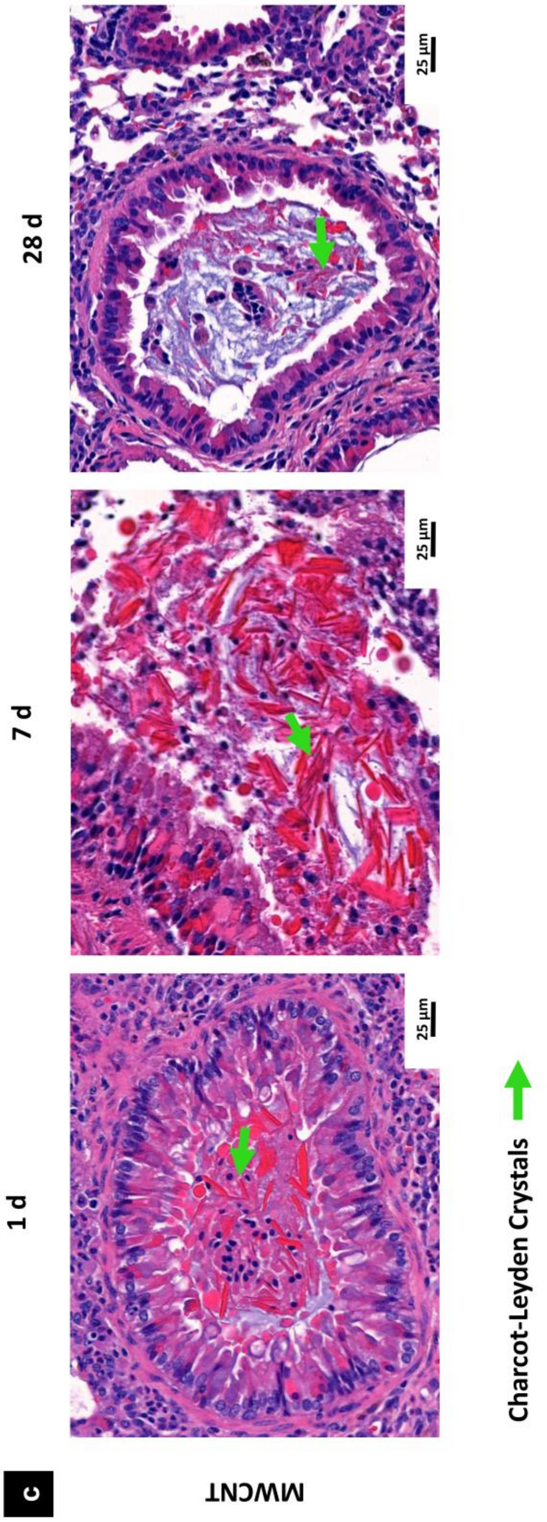

Figure S5. Histopathological analysis of lungs. Mice were exposed by oropharyngeal aspiration to 1 µg (Low dose; (**a**)) or 10 µg (High dose; (**b**)) of nanomaterials, applied three times, over a 28 day period. One, 7, 28, 84 days after the last exposure, lungs were harvested, processed, and stained with H&E. Lung perimeter, pleural and bronchial thicknesses, and the formation of granulomatous (yellow arrows) or bronchus-associated lymphoid tissue (BALT)(blue arrows) structures and Charcot-Leyden crystals (green arrows) were evaluated by optical microscopy (n=3). (**c**) Higher magnification H&E images highlighting the accumulation of

Charcot-Leyden crystals (green arrows) in the bronchial lumen of mice repeatedly exposed to high-dose of MWCNTs.

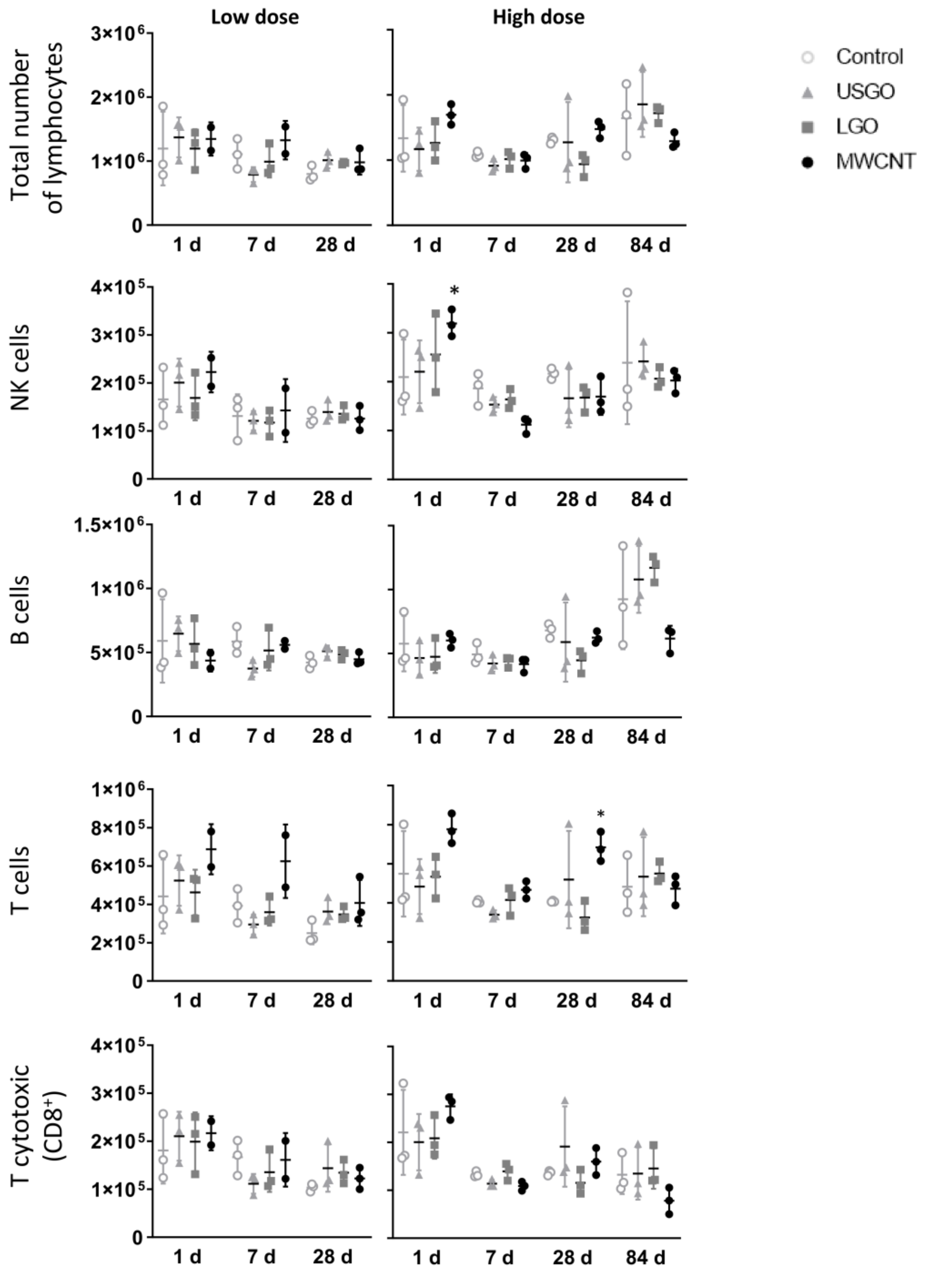

Figure S6. Lymphocyte populations in the whole lung. Mice were exposed by oropharyngeal aspiration to 1 µg (Low dose) or 10 µg (High dose) of nanomaterials, applied three times, over a 28 day period. One, 7, 28, 84 days after the last exposure, left lungs (whole lung w/o lavage) were digested, and then individualised cells were stained with antibodies for phenotyping of lymphocytes by flow cytometry. NK cells were isolated from the others lymphocytes based to their low expression of MHC II, low scattering and intermediate expression of CD11b. T cells were first differentiated based on their high expression of CD3 and then on CD4 or CD8 expression. B cells were isolated based on their high expression of MHC II and specific

expression of CD38 and B220. Two-way ANOVA followed by Dunnett's post-hoc test was used to evaluate difference in the number of lymphocytes compared to the negative control (n=3; p<0.05:*, p<0.01:**, p<0.001:***).

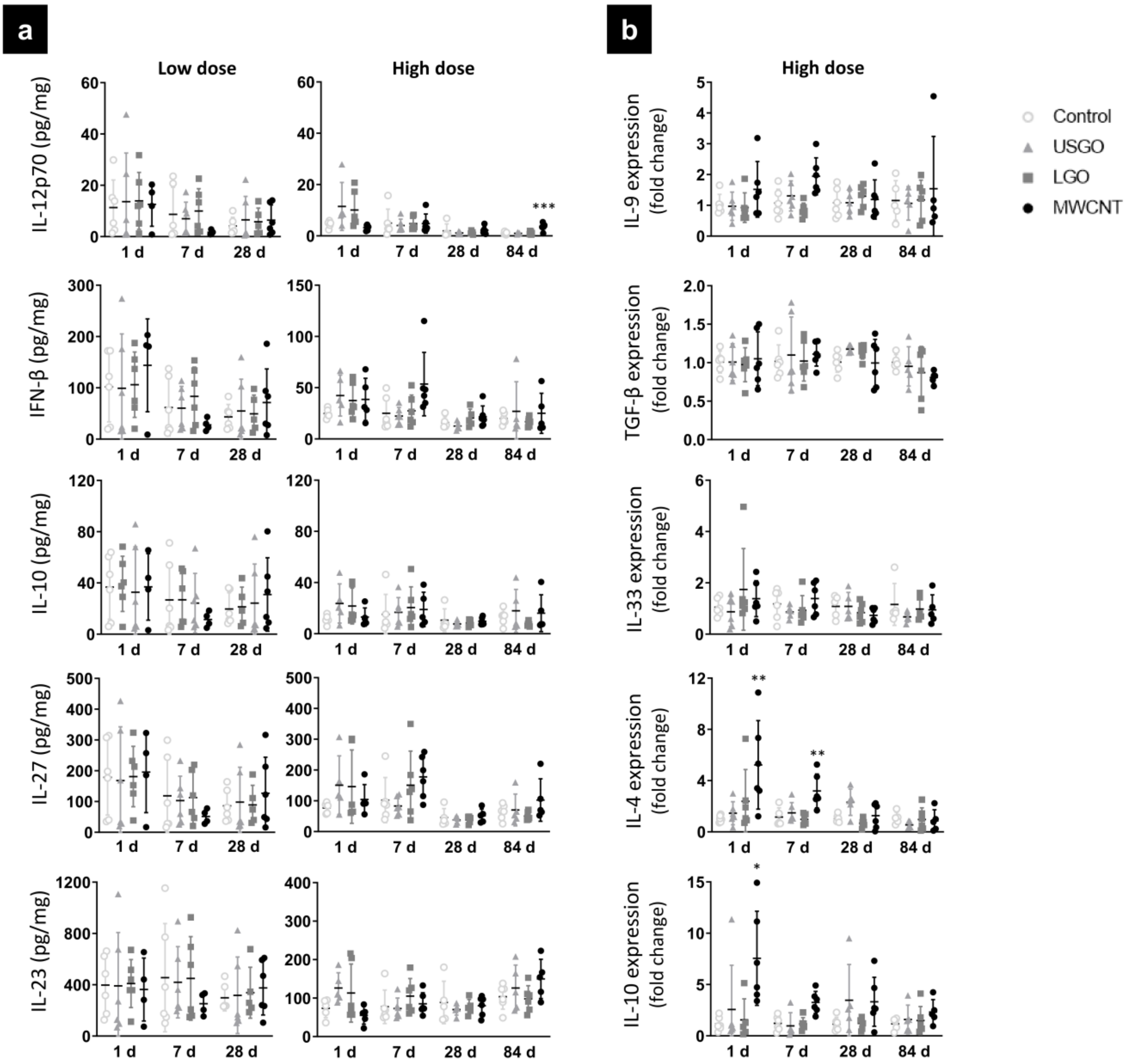

Figure S7. Levels of inflammatory mediators associated to the adaptive immunity in lungs. Mice were exposed by oropharyngeal aspiration to 1 µg (Low dose) or 10 µg (High dose) of nanomaterials, applied three times, over a 28 day period. One, 7, 28, 84 days after the last exposure, lungs were harvested and then lysed to evaluate differences in gene expression by RT-qCPR (**a**) or protein concentration by ELISA (**b**), compared to the negative control. For proteins, each sample was normalized to its own total protein concentration evaluated using a BCA assay. At each time-point, one-way ANOVA followed by Dunnett's post-hoc test or Kruskall-Wallis followed by Dunn's post-hoc test was used to evaluate statistical differences compared to the negative control (n=6; p<0.05:*, p<0.01:**, p<0.001:***).

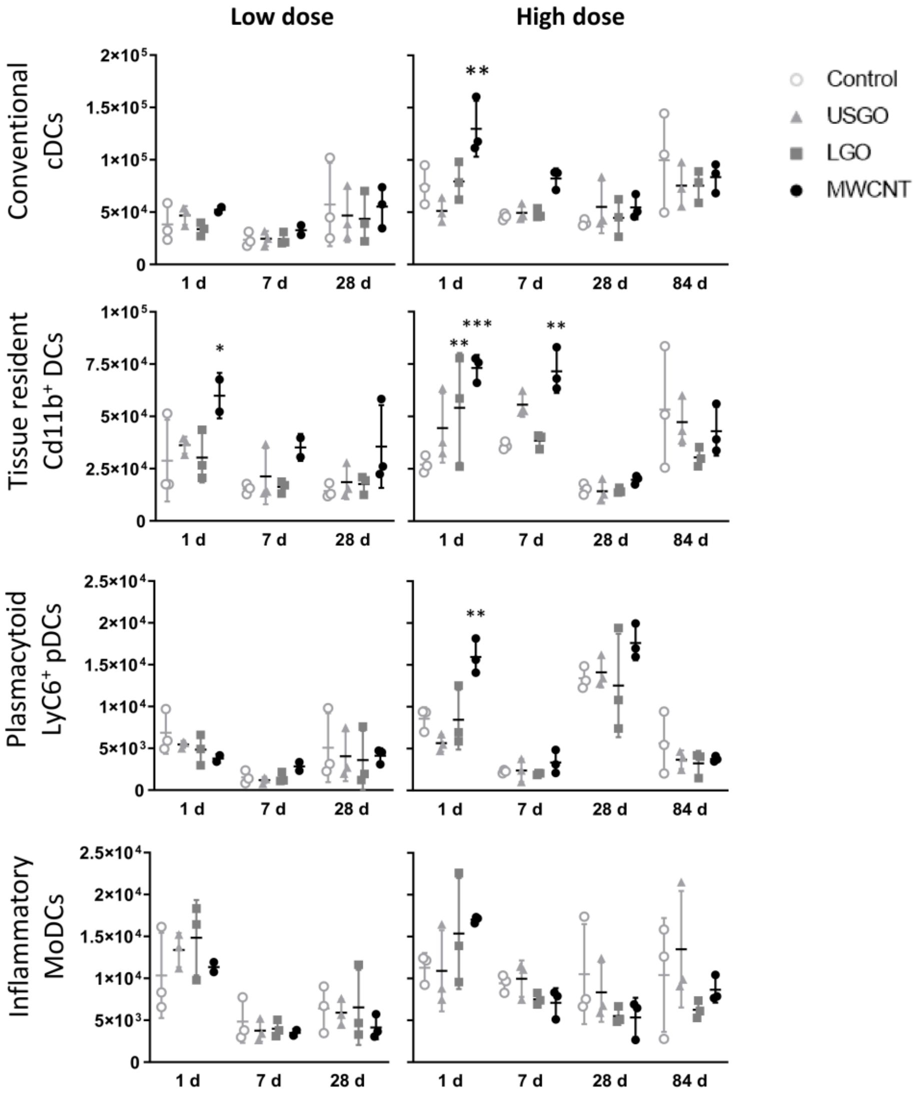

Figure S8. Dendritic cells populations in the whole lung. Mice were exposed by oropharyngeal aspiration to 1 µg (Low dose) or 10 µg (High dose) of nanomaterials, applied three times, over a 28 day period. One, 7, 28, 84 days after the last exposure, left lungs (whole lung w/o lavage) were digested, and individualised cell suspensions were stained with antibodies for phenotyping of dendritic cells by flow cytometry. Dendritic cells (DCs) populations were differentiated according to their expression of MHC II, CD11b and Ly6C. Two-way ANOVA followed by Dunnett's post-hoc test was used to evaluate significant differences in the number of immune cells compared to the negative control (n=3; $p<0.05$:*, $p<0.01$:**, $p<0.001$:***).

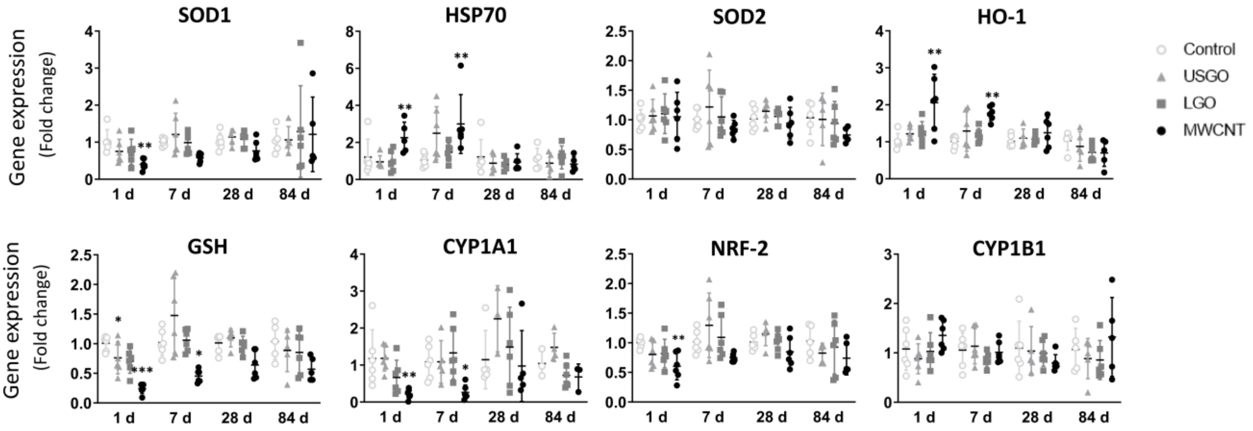

Figure S9. Gene expression of oxidative stress markers in lungs. Mice were exposed by oropharyngeal aspiration to 10 µg (High dose) of nanomaterials, applied three times, over a 28 day period. One, 7, 28, 84 days after the last exposure, lungs were harvested and then lysed to evaluate differences in gene expression compared to the negative control by RT-qCPR. Graphs show individual values, mean and standard deviation. At each time point, one-way ANOVA followed by Dunnett's post-hoc test or Kruskall-Wallis followed by Dunn's post-hoc test was used to evaluate differences of gene expression compared to the negative control (n=6; p<0.05:*, p<0.01:**, p<0.001:***).

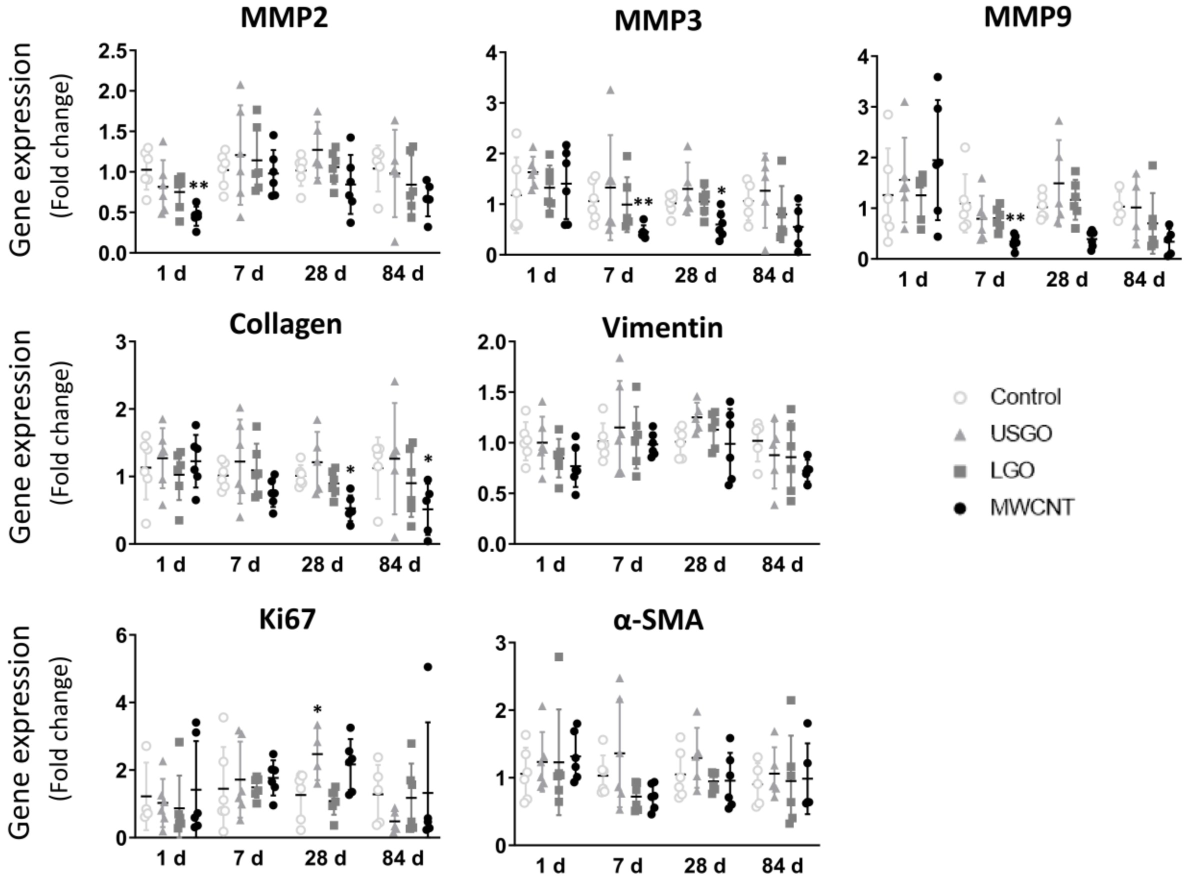

Figure S10. Gene expression of tissue remodelling markers in lungs. Mice were exposed by

oropharyngeal aspiration to 10 µg (High dose) of nanomaterials, applied three times, over a 28 day period. One, 7, 28, 84 days after the last exposure, lungs were harvested and then lysed to evaluate differences in gene expression compared to the negative control by RT-qCPR. Graphs show individual values, mean and standard deviation. At each time point, one-way ANOVA followed by Dunnett's post-hoc test or Kruskall-Wallis followed by Dunn's post-hoc test was used to evaluate difference of gene expression compared to the negative control (n=6; $p<0.05$:*, $p<0.01$:**, $p<0.001$:***).

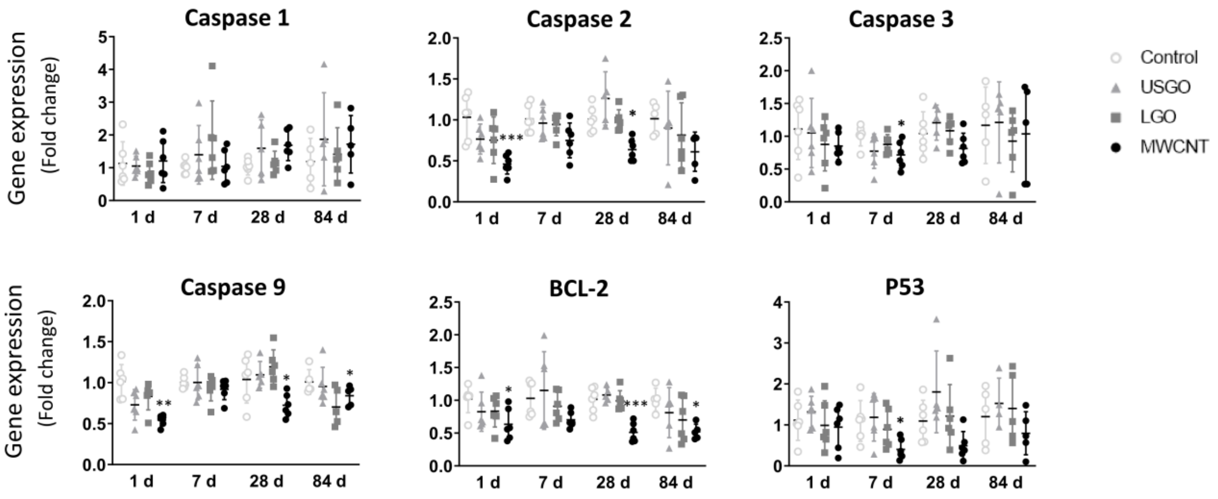

Figure S11. Gene expression of apoptosis markers in lungs. Mice were exposed by oropharyngeal aspiration to 10 µg (High dose) of nanomaterials, applied three times, over a 28 day period. One, 7, 28, 84 days after the last exposure, lungs were harvested and then lysed to evaluate differences in gene expression compared to the negative control by RT-qCPR. Graphs show individual values, mean and standard deviation. At each time point, one-way ANOVA followed by Dunnett's post-hoc test or Kruskall-Wallis followed by Dunn's post-hoc test was used to evaluate differences of gene expression compared to the negative control (n=6; $p<0.05$:*, $p<0.01$:**, $p<0.001$:***).

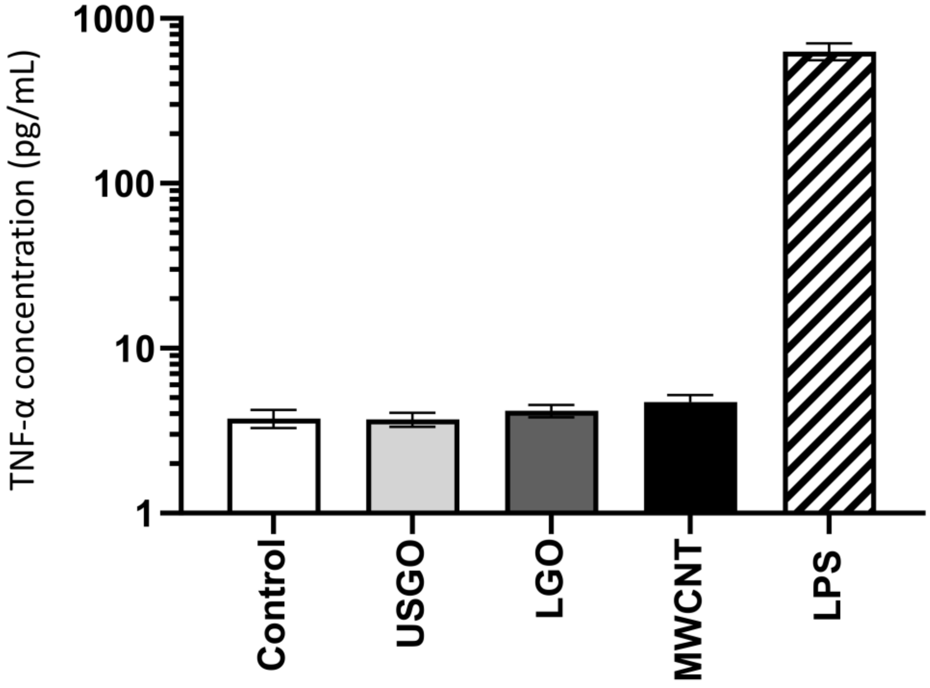

Figure S12. Endotoxin level evaluated in BMDMs. Mice bone marrow derived macrophages were exposed to non-toxic concentrations of USGO and LGO (50 µg/mL) to MWCNT (5 µg/mL) or to vehicle (WFI). LPS at 100 ng/mL was used as positive control of inflammation.

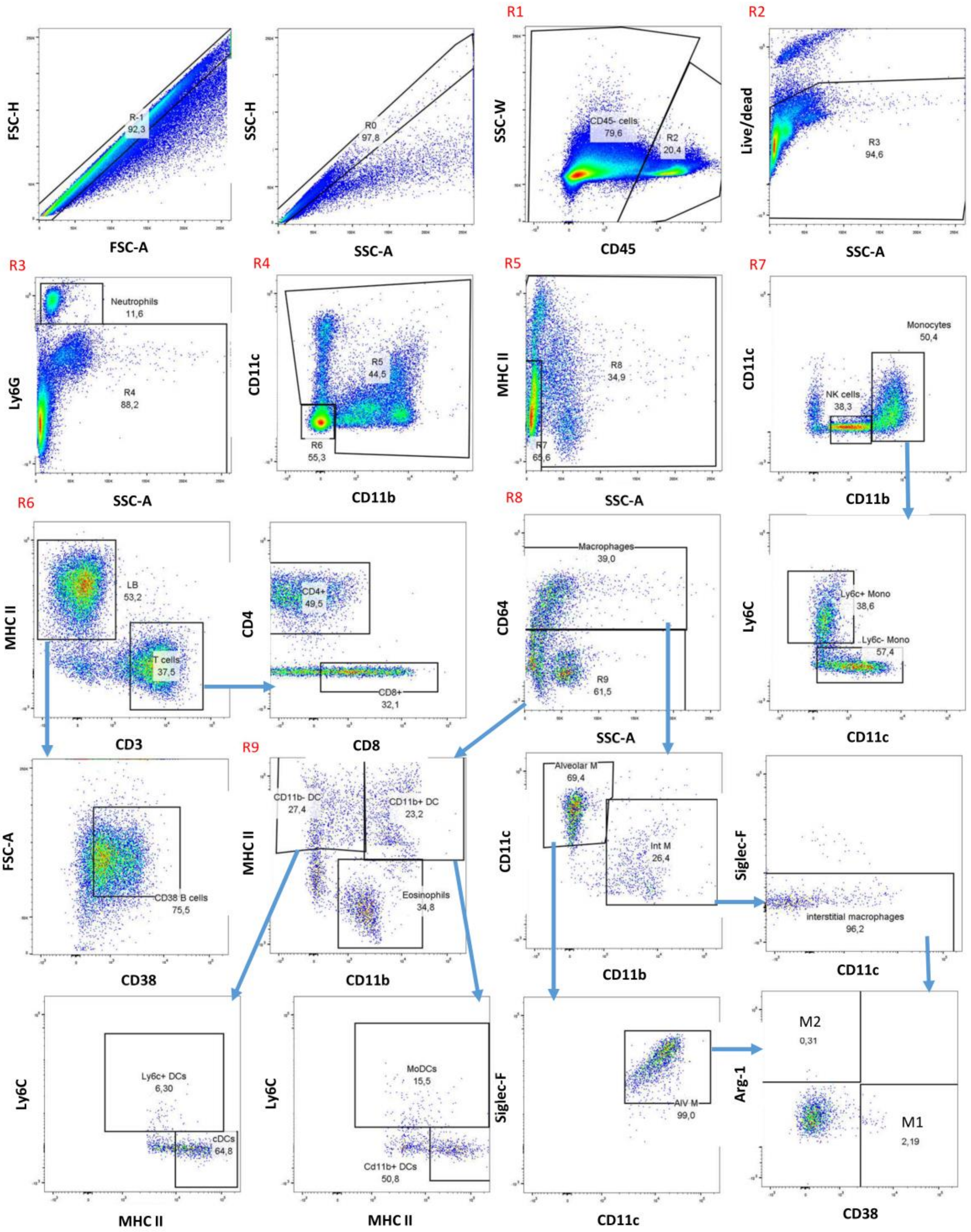

Figure S13. Gating strategy to phenotype immune cells by flow cytometry. Debris and doublets were first excluded using forward (FSC) *vs* side scattering (SSC) plot. Immune cells were differentiated from non-immune cells according to their high CD45 expression (**R1**). Dead Immune cells were then eliminated from the analysis based on their high Live/Dead intensity (**R2**). Neutrophils were identified based on their high expression of Ly6G and intermediate SSC, and were then confirmed to be $CD11b^+$ (**R3**). Arginase expression by neutrophil was also evaluated. Lymphocytes B and T were isolated owing to their Low CD11c and CD11b expression (**R4**). B cells were isolated based on their high MHC II expression and confirmed to be CD38 intermediate/$^+$ and $B220^+$ (**R6**). T cell populations were distinguished according to their high CD3 value and their expression of CD4 or CD8. NK cells were discriminated due to their intermediate

CD11b and low CD11c expression and low scattering. Monocyte populations were differentiated using their high CD11b and low scattering values and according to their Ly6C expression (**R5** and **R7**). Macrophages were isolated based on their high CD64 expression (**R8**). Alveolar macrophages were confirmed to be CD11c$^+$ and Siglec-F$^+$, whereas interstitial macrophages were CD11b intermediate/$^+$ and Siglec-F$^-$. Macrophage activation towards pro-inflammatory (M1) and anti-inflammatory (M2) subsets was evaluated using CD38 (positive for M1) and Arginase-1 (positive for M2) expression. Dendritic cell populations (Conventional: cDCs, Tissue resident: Cd11b$^+$ DCs, Plasmacytoid: LyC6$^+$ pDCs, Inflammatory: MoDCs) were differentiated according to their expression of MHC II, CD11b, and Ly6C. For each sample, data were expressed in cell number by multiplying the percentage of cell population or subset measured by flow cytometry by the total number of cells counted using a haemocytometer.

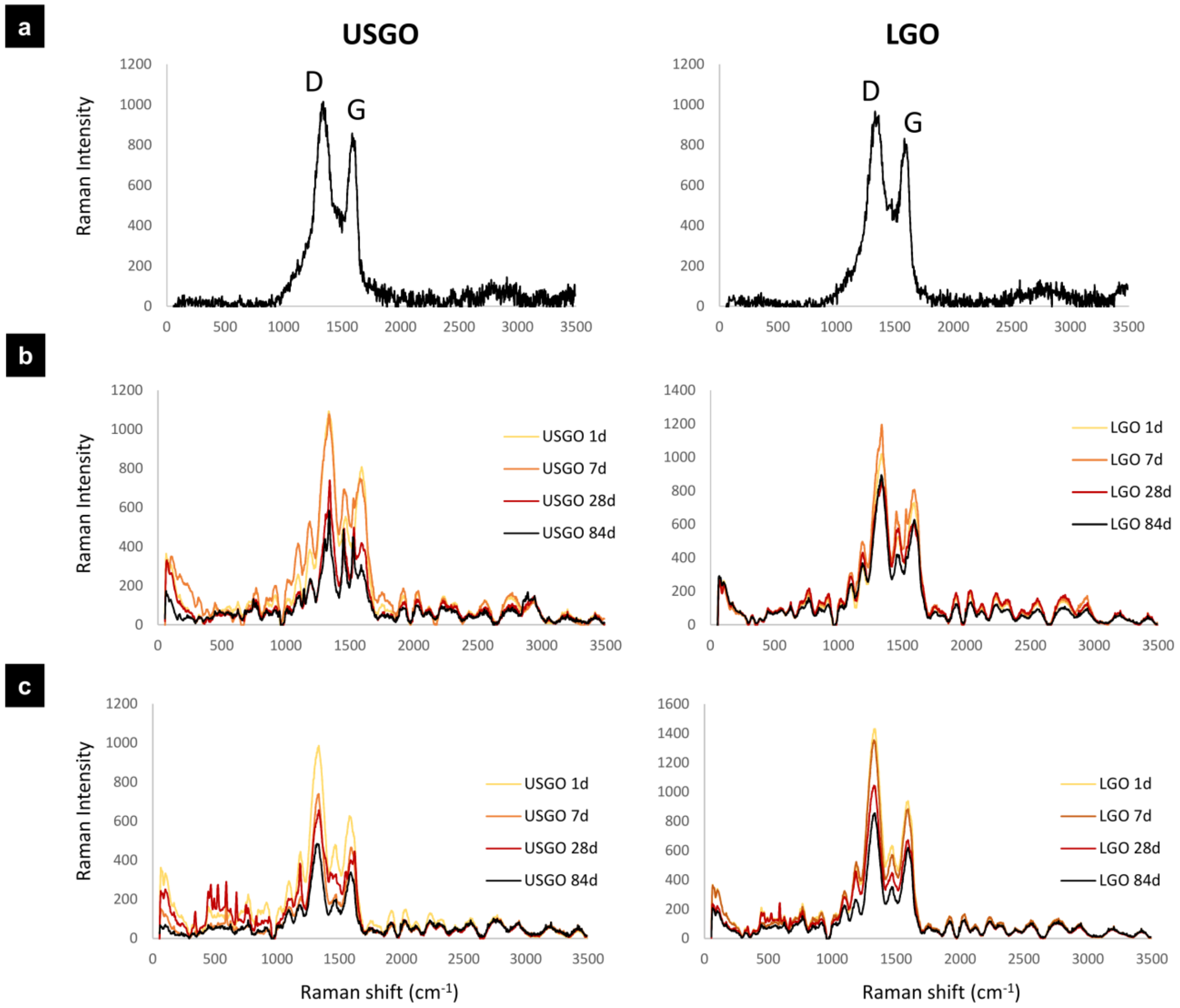

Figure S14. Raman spectra of USGO and LGO. GO was identified based on characteristic D (~1340 cm^{-1}) and G bands (~1580 cm^{-1}). (**a**) Raman signature of bulk USGO and LGO (materials only). (**b**) Raman signature of USGO and LGO in lung tissue at day 1, 7, 28 and 84 after exposure to the high dose. (**c**) Raman signature of USGO and LGO in BAL samples 84 after exposure to the high dose.
